# Active and adaptive *Legionella* CRISPR‐Cas reveals a recurrent challenge to the pathogen

**DOI:** 10.1111/cmi.12586

**Published:** 2016-03-31

**Authors:** Chitong Rao, Cyril Guyard, Carmen Pelaz, Jessica Wasserscheid, Joseph Bondy‐Denomy, Ken Dewar, Alexander W. Ensminger

**Affiliations:** ^1^ Department of Molecular GeneticsUniversity of TorontoTorontoOntarioCanada; ^2^ Public Health OntarioTorontoOntarioCanada; ^3^ Centro Nacional de MicrobiologíaInstituto de Salud Carlos IIIMadridSpain; ^4^ The McGill University and Génome Québec Innovation CentreMontrealQuebecCanada; ^5^ Department of BiochemistryUniversity of TorontoTorontoOntarioCanada; ^6^ Present address: Technology Research Institute BioasterLyonFrance; ^7^ Present address: University of California, San FranciscoSan FranciscoCAUSA

## Abstract

Clustered regularly interspaced short palindromic repeats with CRISPR‐associated gene (CRISPR‐Cas) systems are widely recognized as critical genome defense systems that protect microbes from external threats such as bacteriophage infection. Several isolates of the intracellular pathogen *Legionella pneumophila* possess multiple CRISPR‐Cas systems (type I‐C, type I‐F and type II‐B), yet the targets of these systems remain unknown. With the recent observation that at least one of these systems (II‐B) plays a non‐canonical role in supporting intracellular replication, the possibility remained that these systems are vestigial genome defense systems co‐opted for other purposes. Our data indicate that this is not the case. Using an established plasmid transformation assay, we demonstrate that type I‐C, I‐F and II‐B CRISPR‐Cas provide protection against spacer targets. We observe efficient laboratory acquisition of new spacers under ‘priming’ conditions, in which initially incomplete target elimination leads to the generation of new spacers and ultimate loss of the invasive DNA. Critically, we identify the first known target of *L. pneumophila* CRISPR‐Cas: a 30 kb episome of unknown function whose interbacterial transfer is guarded against by CRISPR‐Cas. We provide evidence that the element can subvert CRISPR‐Cas by mutating its targeted sequences – but that primed spacer acquisition may limit this mechanism of escape. Rather than generally impinging on bacterial fitness, this element drives a host specialization event – with improved fitness in *Acanthamoeba* but a reduced ability to replicate in other hosts and conditions. These observations add to a growing body of evidence that host range restriction can serve as an existential threat to *L. pneumophila* in the wild.

## Introduction

Bacteria are faced with a number of challenges in the environment, including protozoan grazing, bacteriophage infection and the uptake of other selfish DNA that can modulate fitness within a specific niche. Surviving protozoan predation is a critical step in the evolution of intracellular pathogens, with these encounters serving as ‘training grounds’ for subsequent interactions with human phagocytes such as macrophages (Molmeret *et al.*, 2005). When not inside host cell compartments, bacteria must also defend themselves against genome invasion from foreign entities like bacteriophages. One particularly effective source of such protection comes from clustered regularly interspaced short palindromic repeats with CRISPR‐associated genes (CRISPR‐Cas) acquired immune systems (Wiedenheft *et al.*, 2012). Broadly, the main features of CRISPR‐Cas defense are (i) the acquisition of immunity by storing snippets of sequence derived from non‐lethal interactions with foreign invaders and (ii) the use of this stored information in subsequent encounters to cleave an invader's DNA. Immunological memory is stored within an array of identical repeats and non‐identical spacers possessing homology to foreign elements (Heler *et al.*, 2014). A surveillance complex is then generated through the production and packaging of small CRISPR RNA molecules into a ribonucleoprotein CRISPR‐Cas complex, that base pairs with invading nucleic acid upon subsequent exposure and mediates its destruction (van der Oost *et al.*, 2014). To date, several different types of CRISPR‐Cas have been identified, each of which uses distinct protein complexes and mechanisms to cleave targeted DNA (Makarova *et al.*, 2011; Makarova *et al.*, 2015).

For intracellular bacterial pathogens like *Legionella pneumophila*, the true function of CRISPR‐Cas has remained a mystery, due in part to lack of spacer homology with known foreign DNA and a perception that the intracellular lifestyle of these organisms might provide them with some level of innate protection against phage (Sulakvelidze *et al.*, 2001; Broxmeyer *et al.*, 2002). Obfuscating matters further, the most commonly studied strain of *L. pneumophila*, isolated during the eponymous 1976 Philadelphia outbreak of Legionnaires' disease, is itself devoid of CRISPR‐Cas (Chien *et al.*, 2004). At the same time, novel, alternative functions of CRISPR‐Cas systems have been revealed for a number of intracellular pathogens (Ratner *et al.*, 2015): In *Franciscella novicida*, specific components of the type II‐B CRISPR‐Cas system repress the production of a bacterial lipoprotein to evade a host innate immune response (Sampson *et al.*, 2013), whereas in *L. pneumophila* str. 130b, type II‐B Cas2 nuclease activity has been shown to be essential for growth within amoebal hosts (Gunderson and Cianciotto, 2013; Gunderson *et al.*, 2015). Despite these observations that point to a non‐canonical role for *L. pneumophila* CRISPR‐Cas, others have recently noted spacer dynamics consistent with acquisition (Luck *et al.*, 2015). While the targets of *L. pneumophila* CRISPR‐Cas remain undefined, an active and adaptive CRISPR‐Cas system in this organism would mean that its spacers could be viewed as indices reflecting previously invading mobile elements.

*Legionella pneumophila* is a ubiquitous waterborne bacterium that replicates within a diverse set of amoebae and other protists (Rowbotham, 1980; Fields, 1996; Molmeret *et al.*, 2005; Faulkner *et al.*, 2008). Using strategies evolved during these encounters (Gao *et al.*, 1997; Molmeret *et al.*, 2005), the bacteria can also replicate inside alveolar macrophages (Horwitz and Silverstein, 1980) and cause Legionnaires' disease, an often fatal pneumonia (McDade *et al.*, 1977). After replication, *L. pneumophila* spreads through host cell lysis and bacterial egress into the extracellular milieu, providing a window of vulnerability to genomic parasites present in the environment (Isberg *et al.*, 2009). *Legionella pneumophila* genomes display high plasticity (Cazalet *et al.*, 2004; D'Auria *et al.*, 2010; Gomez‐Valero *et al.*, 2011; McAdam *et al.*, 2014; Sanchez‐Buso *et al.*, 2014), much of which is associated with acquired mobile elements, such as conjugative systems that broadly enhance bacterial fitness in the environment (Segal *et al.*, 1999; Glockner *et al.*, 2008; Schroeder *et al.*, 2010; Arambula *et al.*, 2013; Wee *et al.*, 2013; Flynn and Swanson, 2014). The presence of multiple *L. pneumophila* CRISPR‐Cas systems with spacers of unknown origin, however, suggests an ongoing defense against one or more heretofore‐unidentified detrimental exogenous sequences (D'Auria *et al.*, 2010; Faucher and Shuman, 2011). As the historical genomic record of encounters between *L. pneumophila* and parasitic DNA, these spacers hold the potential to provide a much‐needed window into the evolutionary dynamics at play within natural reservoirs of this important pathogen.

## Results

### Identification of a type I‐C CRISPR‐Cas system in the finished genome of L. pneumophila str. Toronto‐2005

**Figure 1:**
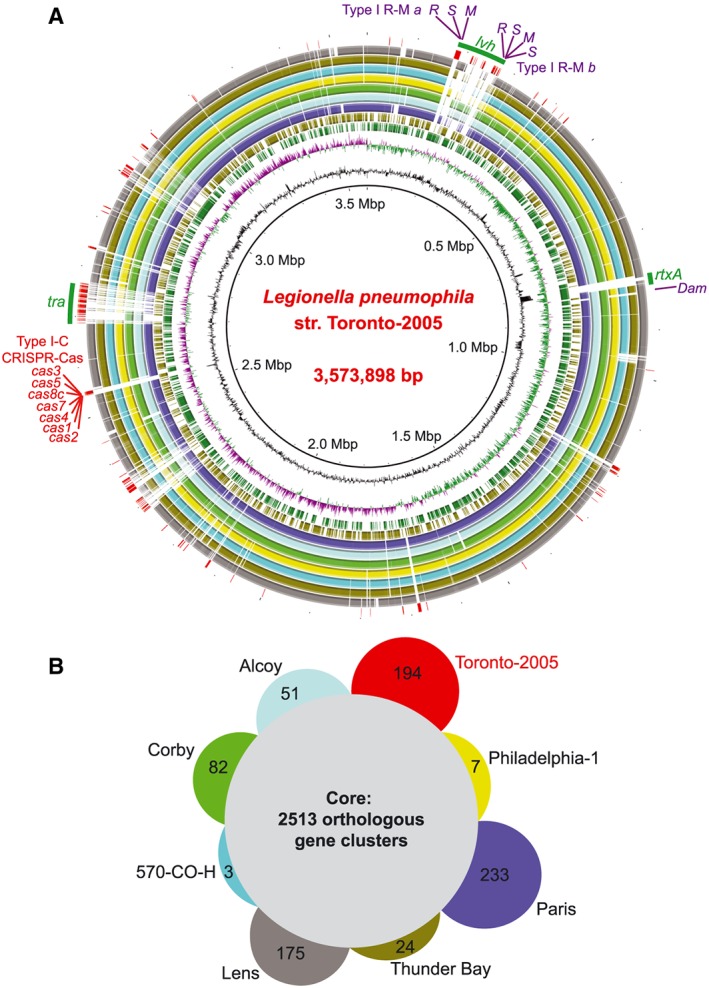
The complete genome of *Legionella pneumophila* str. Toronto‐2005 includes type I‐C CRISPR‐Cas. A. The circularized, finished genome of a ST222 strain that caused a 2005 outbreak of Legionnaires' disease in Toronto. The *L. pneumophila* str. Toronto‐2005 genome contains one circular chromosome of 3 573 898 nt, with an average GC content of 39.6%. The automated annotation pipeline Prokka (Seemann, 2014) detected 3237 genes, including 43 tRNA genes and 3 rRNA operons. Notably, a full sequence of the long, repetitive *rtxA* gene (23 796 bp) was obtained for the first time (Fig. S1A) by taking advantage of long (>10 kb) reads from Pacific Biosciences sequencing. Shown is the genome ring map generated using BLAST Ring Image Generator v0.95 (Alikhan *et al.*, 2011). Circles from innermost to outmost: proportional scale, GC content, GC skew (+ green, ‐ purple), CDS on the sense (green) and antisense (olive) strand, BLAST comparisons with *L. pneumophila* str. Paris, Alcoy, Corby, Philadelphia‐1, 570‐CO‐H, Thunder Bay and Lens genomes (blank regions indicate no sequence with over 50% identity) and genes present in Toronto‐2005 but not in the seven published genomes. Highlighted are the type I‐C CRISPR‐Cas system and the restriction–modification systems in the Toronto‐2005 genome. The methylome contains three restriction–modification methylation motifs: one type II motif, 5′‐G^m6^ ATC‐3′ (Dam), and two type I motifs, 5′‐G^m6^ ARN5CTAA‐3′ and 5′‐AA^m6^AYN6ATGC‐3′. B. A Venn diagram showing the numbers of shared and unique genes in the finished *L. pneumophila* genomes. Orthologous gene clusters across these genomes were identified with PGAP using the MultiParanoid method with default settings (Zhao *et al.*, 2012). Outside a core set of 2513 gene clusters, the pan‐genome includes several strain‐specific loci: *L. pneumophila* str. Toronto‐2005 (194), Philadelphia (7), Paris (233), Lens (175), Alcoy (51), Corby (82), Thunder Bay (24) and 570‐CO‐H (3).

Compelling evidence for canonical CRISPR‐Cas activity in *L. pneumophila* comes from our genomic analysis of several emergent strains of *L. pneumophila* sequence type (ST) 222, isolated in clinical cases in and around Ontario, Canada (Tijet *et al.*, 2010). One of these strains, *L. pneumophila* str. Toronto‐2005, caused an explosive outbreak of disease in Toronto that sickened 135 people, of whom 23 died (Gilmour *et al.*, 2007). Using Pacific Biosciences sequencing, we obtained a gapless, circularized genome for this strain (Fig. 1A), along with the first complete methylome of any *L. pneumophila* isolate.

**Figure 2:**
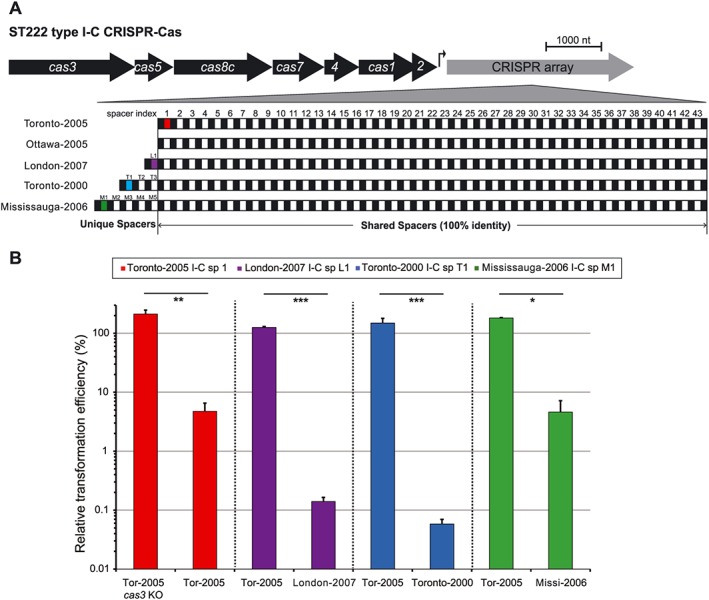
Genome defense by *Legionella pneumophila* type I‐C CRISPR‐Cas. A. Comparison of the type I‐C CRISPR‐Cas systems in five unrelated ST222 strains provides genomic evidence strongly suggestive of CRISPR adaptivity in the environment. An expanded version of the CRISPR array indicates the number of spacers, denoted by white boxes, each flanked by repeats shown with black boxes. Note that three ST222 strains have additional spacers at the leader side of the CRISPR locus. Spacers highlighted by colours correspond to protospacers tested below. B. Individual CRISPR spacers protect against transformation of targeted plasmids in wild‐type strains but not a Δ*cas3* mutant. Plasmids containing either protospacer or scrambled control sequence were electroporated into the indicated strains (refer to Experimental procedures section and Table S5 for more details). The relative transformation efficiency was calculated by normalizing to the transformation efficiency of the control plasmid. Error bars indicate the standard error of the mean of three biological replicates. *P < 0.05, **P < 0.01, ***P < 0.001, according to Student's *t*‐test against relative transformation efficiencies in the indicated strains.

We next compared gene content between *L. pneumophila* str. Toronto‐2005 and seven other finished genomes of *L. pneumophila* (Fig. 1B). In addition to possessing two type I restriction–modification systems (consistent with our Pacific Biosciences methylome data) and unique variants of two common conjugative systems (*lvh* and *tra)* (Fig. S1), the genome includes a novel type I‐C CRISPR‐Cas system (Fig. 1A) (Makarova *et al.*, 2011; Makarova *et al.*, 2015). This system consists of seven *cas* genes (*cas1/2/3/4/5/7/8c*) and a downstream CRISPR array that includes 43 spacers interlaced with the repeat sequence (5′‐GTCGCGCCCCGTGCGGGCGCGTGGATTGAAAC‐3′) (Fig. 2A).

To determine the evolutionary dynamics of CRISPR‐Cas and other unique genetic loci in strains closely related to *L. pneumophila* str. Toronto‐2005, we performed comparative genomic analysis against Illumina‐generated draft genomes of another four ST222 isolates: Toronto‐2000, Ottawa‐2005, Mississauga‐2006 and London‐2007 (Fig. S2A). Notably, the same type I‐C CRISPR‐Cas operon is present in all five strains, and the Cas proteins are 100% identical.

In *L. pneumophila* str. Mississauga‐2006, we also identified a 114 kb plasmid that contains an additional type I‐F CRISPR‐Cas system (Fig. S2B) (Makarova *et al.*, 2011; Makarova *et al.*, 2015). This type I‐F system consists of six *cas* genes (*cas1/3/6f, csy1/2/3*) and a CRISPR array that includes 74 spacers interlaced with the repeat sequence (5′‐GTTCACTGCCGTACAGGCAGCTTAGAAA‐3′). Comparisons to previously identified *L. pneumophila* type I‐F *cas* genes (D'Auria *et al.*, 2010) reveal that the *L. pneumophila* str. Mississauga‐2006 type I‐F system is closely related to a CRISPR‐Cas system on a plasmid from *L. pneumophila* str. Lens – differing by only seven synonymous and eight non‐synonymous polymorphisms over the 8.1 kb gene cluster. Remarkably, despite these nearly identical Cas operons, the downstream CRISPR arrays of the two systems consist of two entirely distinct sets of spacers. One interpretation of this observation is that robust spacer acquisition in one or both of these systems has displaced any ancestral spacers present at the time of their evolutionary divergence. Note that because these plasmid‐based, type I‐F CRISPR‐Cas loci could be transferred horizontally, spacer acquisition within these arrays could have also occurred within completely different bacterial hosts.

### L. pneumophila CRISPR‐Cas is both active and adaptive

While the type I‐C Cas proteins are identical in all five closely related ST222 strains, genomic comparison of their CRISPR arrays reveals evidence of environmental spacer acquisition. CRISPR arrays acquire spacers in a directional fashion, with new spacers located upstream of ancestral spacers within the unprocessed array transcript (Barrangou *et al.*, 2007). Notably, all five arrays share a core set of 43 spacers, with one new spacer in *L. pneumophila* str. London‐2007, three new spacers in *L. pneumophila* str. Toronto‐2000 and five new spacers in *L. pneumophila* str. Mississauga‐2006, all located at the leader side of the CRISPR array (Fig. 2A). We hypothesize that each of these additional spacers was acquired after the divergence from a common ST222 ancestor possessing the core 43 spacers present in *L. pneumophila* str. Toronto‐2005 and Ottawa‐2005. In addition to being the simplest explanation for the spacer differences that we observe, this hypothesis is also consistent with the whole‐genome phylogeny between these five strains (Fig. S2C).

Our data also show that several *cas* genes, along with the CRISPR RNA, are up‐regulated during post‐exponential growth (Fig. S3A), a growth phase that mimics the condition immediately prior to *L. pneumophila* lysis from the host cell and its transit through the extracellular environment (Byrne and Swanson, 1998). We next asked if these systems were indeed active (able to defend against protospacer‐containing foreign DNA). To experimentally test the activity of the type I‐C CRISPR‐Cas system, we performed an established transformation efficiency assay (Marraffini and Sontheimer, 2008) using plasmids containing either (i) protospacers that match CRISPR spacers or (ii) a scrambled untargeted sequence. Importantly, normalizing to this scrambled plasmid controls for any strain‐to‐strain differences in overall transformation efficiencies (Bondy‐Denomy *et al.*, 2013). Using this approach, we showed a significant reduction in transformation efficiency of CRISPR‐targeted plasmids in *L. pneumophila* str. Toronto‐2005 (Fig. 2B). Similar with other type I CRISPR‐Cas (Brouns *et al.*, 2008); (Westra *et al.*, 2012), this CRISPR activity is Cas3‐dependent as no transformation inhibition was observed in a Δ*cas3* mutant (Fig. 2B). Notably, spacers located along the entire length of the repeat‐spacer array were able to protect against protospacer‐containing sequences, although we observe a trend of decreasing inhibition efficiency for spacers further from the transcriptional start of the array (Fig. S3B). We also tested the newly acquired spacers in the other type I‐C CRISPR arrays (Fig. 2A) and observed protection against each of their targets relative to the scrambled control (Fig. 2B).

### Functional type I‐C spacer acquisition occurs rapidly in the presence of foreign DNA

While we observed 10‐ to 10^3^‐fold protection by the type I‐C systems, we hypothesized that any instances of incomplete elimination of protospacer‐containing transformants in our earlier assays might establish conditions favourable to the acquisition of secondary spacers targeting the plasmid backbone. First, we used Sanger sequencing to confirm that our infrequent transformants recovered after electroporation with a plasmid (pSp1) targeted by spacer‐1 did not have spacer or plasmid mutations that might explain their recovery on selective media (data not shown). This ‘incomplete’ CRISPR defense in the assay is consistent with previous observations in *Pseudomonas aeruginosa* (Cady *et al.*, 2012; Bondy‐Denomy *et al.*, 2013; Pawluk *et al.*, 2014) and suggests that high‐copy targeted plasmids are able to persist (if only temporarily and at a much reduced frequency) in the presence of CRISPR‐Cas activity. To test this interpretation of our assays, we first transformed both wild type and Δ*cas3* transformants with the spacer‐1 plasmid. Starting with these recovered transformants, we used a liquid handling system to automatically passage both strains for 20 generations – monitoring each population over time for plasmid loss by plating on selective and non‐selective media (Fig. S4). Consistent with active but incomplete defense against the high‐copy plasmid, we observed plasmid maintenance in the Δ*cas3* background but gradual plasmid loss in the wild‐type strain.

**Figure 3:**
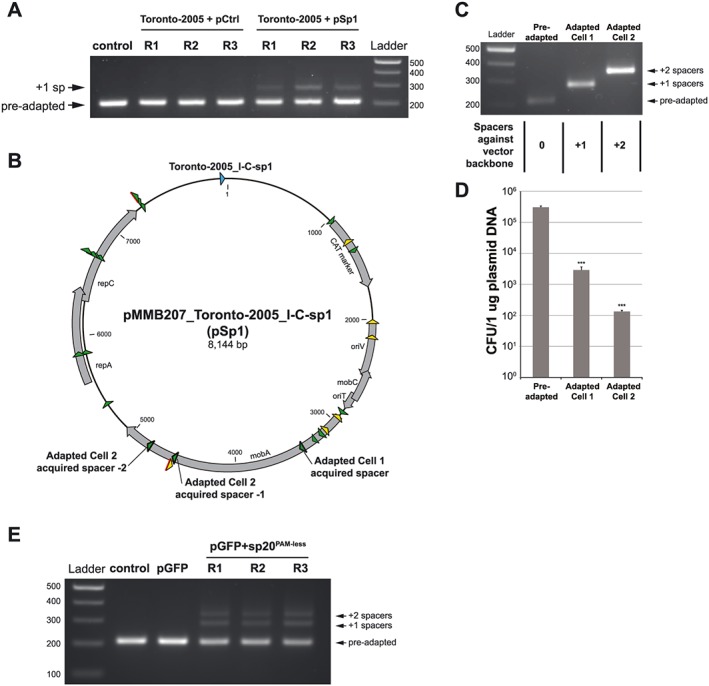
*Legionella pneumophila* type I‐C CRISPR‐Cas is hyperactive in spacer acquisition under priming conditions. A. Spacer acquisition during laboratory passage. *Legionella pneumophila* str. Toronto‐2005 was transformed with either non‐targeted control plasmid pCtrl or priming plasmid pSp1 (containing the protospacer for Toronto‐2005_I‐C‐sp1). The transformants, each with three biological replicates (R1‐R3), were passaged in AYE broth in the absence of antibiotic selection for 20 generations. Shown are the PCR products of the leader side of the CRISPR array amplified from the pre‐adapted strain (control) and each of the passaged cultures. Higher molecular weight bands suggest putative spacer acquisition events, as one additional repeat‐spacer unit adds ~67 bp to the leader side of the CRISPR array. B. Distribution of new spacers acquired in *L. pneumophila* str. Toronto‐2005 mapped to the pMMB207_Toronto‐2005_I‐C‐sp1 (pSp1) priming plasmid. Twenty‐three independently acquired spacers target the plasmid backbone and are indicated by green (protospacers located in one strand) and yellow (protospacers located in the other strand) arrowheads. (Note that two independently acquired spacers target the same protospacer sequence on the plasmid.) Protospacers have a 5′‐GAA‐3′ PAM, except the two marked in red that contain other PAM sequences (refer to Table S1B). C. Two clones of *L. pneumophila* str. Toronto‐2005 after priming were found to have lost the plasmid and gained one and two additional spacers, respectively, that target the pMMB207 plasmid backbone (refer to ‘Adapted Cell’ protospacers in B). D. Each of these adapted strains was able to defend against the control plasmid that originally was not targeted. Shown is the transformation efficiency calculated as colony‐forming units per 1 µg plasmid. Error bars indicate the standard error of the mean of three electroporations. ***P < 0.001, according to Student's *t*‐test against the transformation efficiency in the pre‐adapted strain. E. Another non‐targeted plasmid pGFP was modified to include PAM‐less Toronto‐2005_I‐C‐sp20 sequence to generate a priming plasmid pGFP‐sp20. Following the same approach as in A, this pair of plasmids was used to directly test the effect of priming on spacer acquisition under laboratory conditions.

We next asked whether these growth conditions would select for the acquisition of new spacers. We passaged the wild‐type strain with either targeted (pSp1) or untargeted (pCtrl) plasmids for 20–40 generations using the same approach as described in the preceding texts. Afterwards, we PCR amplified the leader side of the CRISPR array in each population and observed the presence of higher molecular weight bands in each of the pSp1 cultures but in none of the pCtrl transformants (Fig. 3A). To confirm that these observations reflected the acquisition of novel spacer sequences, we used Sanger sequencing to identify 22 unique spacers – all with homology to the vector backbone of pSp1 (Fig. 3B, Table S1B). Notably, 20/22 of these spacers match locations on the plasmid with a 5′‐GAA‐3′ protospacer adjacent motif (PAM) sequence immediately downstream – the same PAM that we predicted based on other type I‐C systems (Mojica *et al.*, 2009) and used in our earlier transformation assays (Fig. 2B).

To test whether these newly acquired spacers provided additional CRISPR‐Cas defense, we screened 48 colonies of the pSp1 passaged population by PCR and identified two *L. pneumophila* isolates with one and two extra novel spacers respectively (Fig. 3C, refer to Fig. 3B, Table S1B for spacer identities of each strain). Given that these newly acquired spacers target the shared vector backbone of pSp1 and pCtrl, we next measured the transformation efficiency of the previously untargeted pCtrl in each of these adapted isolates and compared them to results with the ancestral strain from which they were derived (Fig. 3D). Each adapted isolate displayed 10^2^‐ to 10^3^‐fold protection against the control plasmid, indicating that these new spacers were active in each adapted isolate.

Spacer acquisition can be accelerated through a process that relies on recruitment of the CRISPR‐Cas complex to an imperfect target, a phenomenon known as ‘priming’ (Datsenko *et al.*, 2012; Fineran *et al.*, 2014; Heler *et al.*, 2014). Having observed robust spacer acquisition by passaging *L. pneumophila* str. Toronto‐2005 with the targeted plasmid (pSp1), we next asked whether our observations were analogous to the priming observed previously in other systems (Datsenko *et al.*, 2012; Fineran *et al.*, 2014; Li *et al.*, 2014; Richter *et al.*, 2014). To that end, we passaged transformants containing either a green fluorescent protein (GFP)‐expressing plasmid (‘unprimed’) or the same plasmid with a PAM‐less protospacer sequence downstream of GFP (‘primed’). After 20 generations of passage, we observed robust, multiple spacer acquisition in populations with the PAM‐less protospacer plasmid (Fig. 3E) but not those passaged with the pGFP control. Collectively, these data suggest that the type I‐C CRISPR‐Cas system is robustly adaptive under priming conditions.

### Numerous L. pneumophila CRISPR‐Cas systems contain spacers matching the same genetic element (LME‐1)

**Figure 4:**
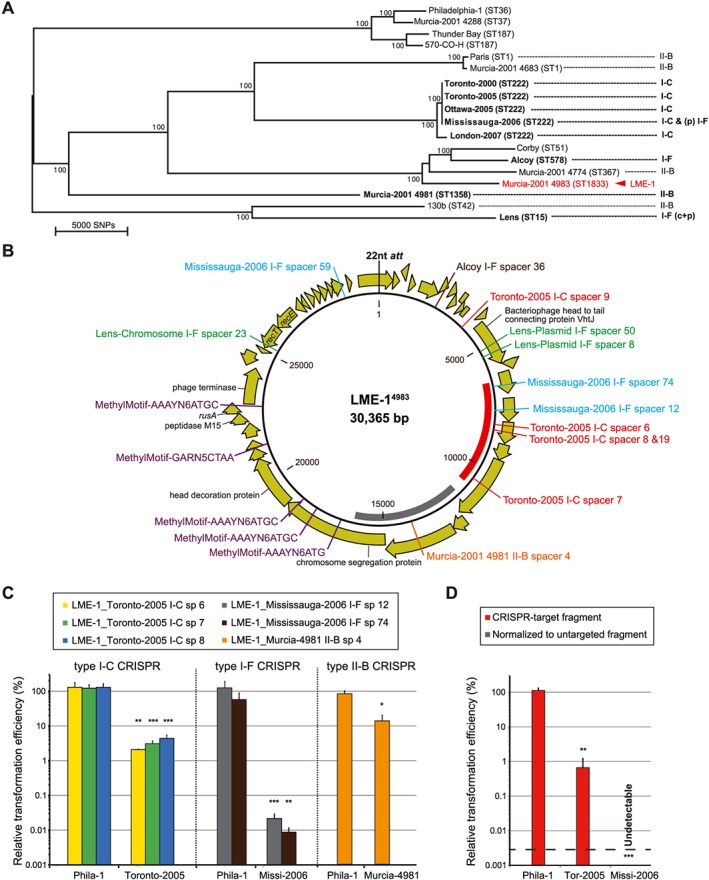
Multiple *Legionella pneumophila* CRISPR‐Cas systems target the same 30 kb sequence element. A. CRISPR‐Cas systems are widespread in *L. pneumophila*. Shown is the core genome‐based neighbour‐joining phylogenetic tree of previously published *L. pneumophila* genomes and ten genomes newly sequenced in this study. The sequence type of each strain is given, with the CRISPR‐Cas system(s) in each strain indicated as well. Note that the environmental strain *L. pneumophila* str. Murcia‐4983 harbours a 30 kb element (LME‐1) that is targeted by multiple *L. pneumophila* CRISPR‐Cas systems (highlighted in bold). B. Several *L. pneumophila* CRISPR‐Cas systems target LME‐1^4983^, often using multiple spacers. The element is 30 365 bp in length and contains 41 ORFs predicted by Prokka (Seemann, 2014). Similar to an orthologous element previously described in *L. pneumophila* str. RC1 (Luneberg *et al.*, 2001), LME‐1^4983^ contains proteins related to bacteriophage and DNA recombination. Highlighted are the sequences targeted by *L. pneumophila* CRISPR spacers (and the Toronto‐2005 type I R‐M systems). C. Distinct *L. pneumophila* CRISPR‐Cas systems protect against individual protospacers in LME‐1^4983^. Plasmids containing indicated sequences (with flanking tri‐nucleotides) from LME‐1^4983^ were electroporated into the indicated strains. The relative transformation efficiency was calculated by normalizing to the transformation efficiency of the control plasmid that contains an untargeted sequence. D. The type I‐C and type I‐F CRISPR‐Cas systems in *L. pneumophila* ST222 strains protect against transformation of LME‐1 fragments. Plasmids containing ~4.5 kb LME‐1 fragments colour coded in B were electroporated into the indicated strains. The relative transformation efficiencies of the CRISPR‐target fragment were calculated by normalizing to that of the untargeted control fragment (grey). Note that *L. pneumophila* str. Mississauga‐2006 has an extra type I‐F CRISPR‐Cas system relative to *L. pneumophila* str. Toronto‐2005. Error bars indicate the standard error of the mean of three biological replicates. *P < 0.05, **P < 0.01, ***P < 0.001, according to Student's *t*‐test against *L. pneumophila* str. Philadephila‐1 relative transformation efficiencies.

Having established that *L. pneumophila* CRISPR‐Cas provides active and adaptive genome defense in the laboratory, we next sought to identify ‘real‐world’ sequence elements that each system is actively protecting against. To increase the number of spacers with which to query, we used CRISPRFinder (Grissa *et al.*, 2007) to search for CRISPR arrays across 12 different sequence types of *L. pneumophila* (Fig. 4A). These analyses indicate that the lack of CRISPR‐Cas in *L. pneumophila* str. Philadelphia‐1 and a handful of closely related strains are exceptions rather than the rule, suggesting an important role for these loci in the normal life cycle of most isolates of the pathogen. Given that many details of the environmental life cycle of *L. pneumophila* remain undefined, spacer identity might provide a much‐needed window into its natural reservoirs.

In total, there are 440 spacers across all three distinct *L. pneumophila* CRISPR‐Cas systems (type I‐C, type I‐F and type II‐B), suggesting a diverse set of environmental challenges encountered by these strains (Table S1A). Bona fide CRISPR‐Cas targets are expected to have (i) complete or nearly complete sequence homology to a 7 nt seed sequence (Semenova *et al.*, 2011), located at the 5′ end (positions 1–5, 7 and 8) of each spacer, (ii) complete or nearly complete homology across the rest of the spacer and (iii) a PAM sequence immediately adjacent to the sequence targeted by the spacer. PAM sequences are specific to each type of CRISPR‐Cas system, where they play a critical role in preventing self‐targeting of the array and other endogenous sequences in the genome (Mojica *et al.*, 2009). We used the CRISPRTarget (Biswas *et al.*, 2013) search tool to identify a list of 40 potential hits for *L. pneumophila* CRISPR‐Cas, each with an appropriate PAM sequence (Table S2).

Predicted *L. pneumophila* CRISPR‐Cas targets with multiple hits include metagenome sequences from activated sludge (More *et al.*, 2014) and marine sources (Venter *et al.*, 2004), yet the incorporeal nature of these sequences currently prevents further investigation into their relevance to *L. pneumophila* biology. Remarkably, however, we predict that numerous CRISPR‐Cas spacers from a set of geographically disparate, evolutionarily distinct strains share the same target: a 30 kb unstable genetic element previously identified in *L. pneumophila* str. RC1 (Luneberg *et al.*, 2001) (Table S2).

The 30 kb element in *L. pneumophila* str. RC1 [referred to hereafter as *Legionella* mobile element‐1^RC1^ (LME‐1^RC1^)] is targeted by six type I‐C CRISPR spacers, seven type I‐F CRISPR spacers and one type II‐B CRISPR spacer, most with zero or one mismatch and a proper PAM. LME‐1^RC1^ was previously isolated from a mutant strain that showed an altered lipopolysaccharide and reduced virulence in human macrophages and guinea pigs (Luneberg *et al.*, 1998). It was later shown that these phenotypes correlate with LME‐1's excision from the bacterial chromosome and existence as a high‐copy episome and, through Southern blot analysis, that other isolates were likely carriers of this episome (Luneberg *et al.*, 2001).

As one or more matches to LME‐1^RC1^ are present in the majority of *L. pneumophila* CRISPR arrays, we reasoned that this element represents a recurrent, widespread challenge to *L. pneumophila* in the environment – and should therefore be discoverable in our laboratory's collection of *L. pneumophila* genomic data. By examining the genomes of a diverse collection of *L. pneumophila* strains from Ontario, the USA and Europe, we identified a Spanish CRISPR‐deficient environmental strain (Murcia‐4983) (Garcia‐Fulgueiras *et al.*, 2003) with Illumina reads homologous to LME‐1^RC1^. *De novo* assembly of these reads produced a contig containing a complete orthologous 30.4 kb element (LME‐1^4983^) (Fig. 4B). LME‐1^4983^ harbours 29 out of 30 predicted LME‐1^RC1^ genes (Luneberg *et al.*, 2001) (with an average amino acid identity of 92%). LME‐1^4983^ also has approximately 1 kb of additional sequence not present in LME‐1^RC1^, within which we identify an additional match by an *L. pneumophila* str. Alcoy CRISPR spacer (Table S3).

Through the transformation efficiency assay described in the preceding texts, we demonstrated that type I‐C, type I‐F and type II‐B CRISPR‐Cas systems with LME‐1 spacers were able to defend against their respective protospacers (Fig. 4C). Importantly, when plasmids containing multiple protospacer sequences were used in the same assay, even greater protection was observed – reaching below the limit of detection in *L. pneumophila* str. Mississauga‐2006 (Fig. 4D).

### Episomal LME‐1 leads to condition‐specific fitness effects

The presence of several independent CRISPR‐Cas spacers targeting LME‐1 indicates that exposure to this element is a common, recurrent feature of *L. pneumophila* environmental persistence. Previous observations that this element could modulate several bacterial phenotypes (modified lipopolysaccharide, reduced replication in macrophages, attenuated virulence in guinea pigs) indicated that the greatest influence on bacterial hosts occurred when LME‐1^RC1^ was excised from the chromosome, where it existed as a high‐copy episome (Luneberg *et al.*, 2001). The isolation of LME‐1^4983^ from our contemporary collection of *L. pneumophila* isolates provided us an opportunity to revisit some of these observations, using approaches unavailable at the time of the initial description of LME‐1^RC1^.

**Figure 5:**
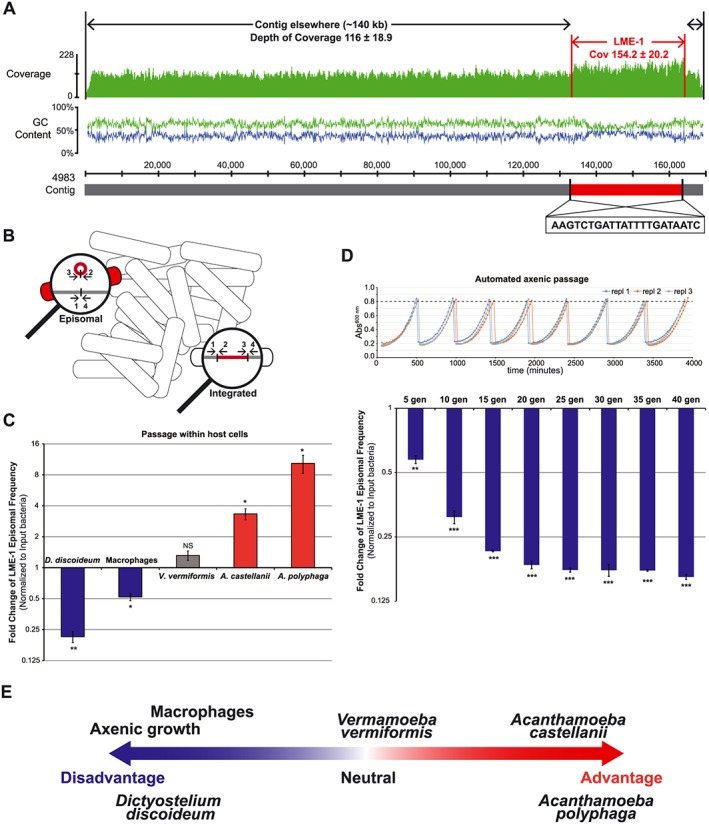
Episomal LME‐1 leads to condition‐specific fitness effects. A. Genomic evidence of episomal LME‐1. The LME‐1 region in the Murcia‐4983 genome has a differential GC content (40.3%, as compared with 36.4% elsewhere in the draft contig) and a higher depth of coverage (~1.33× relative to elsewhere in the contig). Illumina reads were aligned to the contig (~170 kb size), and the depth of coverage along its entire length is displayed. The LME‐1 region is highlighted in red, with the flanking 22 nt *att* sequence identified. B. The two states of LME‐1 (excised episomal state and integrated state) can be quantified in a wild‐type bacterial population using primers flanking the *att* site. Primers: 1‐chrF, 2‐lmeR, 3‐lmeF, 4‐chrR (refer to Experimental procedures section). C and D. Fold change of the frequency of episomal LME‐1 in *Legionella pneumophila* str. Murcia‐4983 after 3 days of intracellular growth in different host types or defined numbers of generations in axenic passage, as measured by qPCR using primers shown in B on genomic DNA extracted directly from bacteria before and after treatment. Error bars indicate the standard error of the mean of three biological replicates, and shown is one representative of two independent experiments. *P < 0.05, **P < 0.01, ***P < 0.001, NS not significant, according to Student's *t*‐test against the input bacteria. Growth curves of *L. pneumophila* str. Murcia‐4983 during axenic passage were also plotted, based on optical density at 600 nm measured in a 48‐well plate (refer to Experimental procedures section for details). E. Episomal LME‐1 leads to condition‐specific fitness effects. Shown is a schematic summary of data in C and D.

First, we examined our original Illumina data for evidence that LME‐1^4983^, like LME‐1^RC1^, might also exist as a high‐copy episomal element in a subpopulation of bacteria. Like LME‐1^RC1^, the LME‐1^4983^ region is flanked by the 22 nt attachment (*att*) sequence (5′‐AAGTCTGATTATTTTGATAATC‐3′) (Luneberg *et al.*, 2001) (Fig. 5A). To detect episomal LME‐1, we designed PCR primers that flank this sequence, thereby distinguishing excised LME‐1 from its integrated form (Fig. 5B). We detected episomal LME‐1 in a population of bacteria grown in rich media and performed Sanger sequencing to confirm recombination at the predicted *att* site (data not shown). Through a quantitative PCR assay (refer to Experimental procedures section), we measure the frequency of the LME‐1 excision after overnight culture in axenic broth to be between 0.41 and 2.19% (Table S6). We used this same assay to estimate that, when episomal, LME‐1 maintains a copy number of over 200 in this wild‐type population (Table S6), which is consistent with previous estimates of LME‐1^RC1^ copy number in episomally enriched mutant strains (Luneberg *et al.*, 2001).

While it was previously shown that an episomally enriched LME‐1^RC1^ mutant caused a bacterial defect in intracellular replication in human HL‐60 cells (Luneberg *et al.*, 2001), human hosts are an evolutionarily dead end to *L. pneumophila*, making such an effect irrelevant to bacterial persistence in the environment (Ensminger *et al.*, 2012). The existence of a ‘selfish’ DNA element frequently targeted by *L. pneumophila* CRISPR‐Cas is, at first glance, paradoxical: Under some evolutionarily relevant conditions, the element must be able to persist, whereas in others, it must cause enough of a fitness defect to justify its frequent targeting by CRISPR‐Cas. To address this, we next measured the effect of LME‐1 on the replication of *L. pneumophila* in human macrophages, four protozoan hosts and one extracellular condition.

While the prior phenotypic characterization of LME‐1^RC1^ was performed using spontaneous mutant strains in which the element was locked in a predominantly episomal form (Luneberg *et al.*, 2001), we decided to determine how the episomal ratio in a *wild‐type* isolate is influenced by the intracellular environment of different hosts. Using the quantitative PCR assay described in the preceding texts, we measured the relative ratio of episomal to integrated LME‐1 in an *L. pneumophila* str. Murcia‐4983 population after passage in both human macrophages and natural hosts (Fig. 5B). Consistent with earlier reports of a defect in macrophages for episomally enriched populations of *L. pneumophila* str. RC1, we observed a significant reduction in LME‐1 episomal frequency in *L. pneumophila* str. Murcia‐4983 after passage through human macrophages (Fig. 5C, Table S6). In stark contrast, passage of *L. pneumophila* through two related protozoan species, *Acanthamoeba castellanii* and *Acanthamoeba polyphaga*, led to an *increase* in episomal bacteria, consistent with our model that one or more environmental conditions should select for LME‐1 maintenance. Indicative of host‐specific effects, growth in another amoebae, *Vermamoeba* (*Hartmannella*) *vermiformis*, led to insignificant changes in episomal frequency (Fig. 5C, Table S6). Strikingly, we observed a significant *disadvantage* of episomal bacteria during replication in *Dictyostelium discoideum*, a different amoebal host of the pathogen (Fig. 5C, Table S6). Extracellular replication of the bacteria also strongly selected against the episome during passage (Fig. 5D). Taken together, these results support a model in which episomal LME‐1 causes condition‐specific fitness effects, with CRISPR‐Cas defenses protecting the broad host range of the pathogen – a feature that is essential for its persistence in most environments (O'Connor *et al.*, 2011; Ensminger *et al.*, 2012; Ensminger, 2016) (Fig. 5E).

### Robust protection against strain‐to‐strain LME‐1 transfer by CRISPR‐Cas

**Figure 6:**
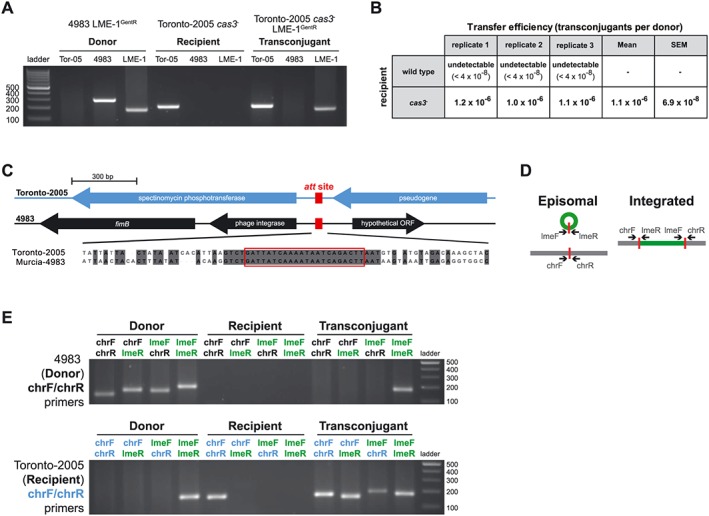
Vulnerability to LME‐1 acquisition in the absence of CRISPR‐Cas defense. A. Transfer of LME‐1 to an *Legionella pneumophila* str. Toronto‐2005 *Δcas3* mutant. In LME‐1 transfer experiments, donor, recipient and transconjugant clones can be distinguished based on the presence or absence of a set of PCR products. As *L. pneumophila* str. Toronto‐2005 and Murcia‐4983 have different gene contents, primers that amplify the Toronto‐2005 ortholog of lpg1151 and the Murcia‐4983 ortholog of lpg2464, along with LME‐1 specific primers, were used to differentiate the three strains and to screen for true transconjugants. Shown is the 2% agarose gel with PCR products from a representative transconjugant clone. B. Quantification of LME‐1 transconjugants in wild‐type and Δ*cas3* recipients. LME‐1 transfer is detected in the Δ*cas3* mutant but not wild‐type strain of Toronto‐2005 as recipient. The transfer efficiency to each recipient was quantified based on the screening described in A and calculated as the number of transconjugants per donor cell. C. *L. pneumophila* str. Toronto‐2005 and Murcia‐4983 have a conserved *att* site for LME‐1 integration (highlighted in the red box) but disparate flanking sequences. D. Taking advantage of these unique flanking sequences, differential chromosomal primers (chrF, chrR) were designed to detect LME‐1 integration in Toronto‐2005 Δ*cas3* mutant and Murcia‐4983. E. LME‐1 integration into the predicted *att* site in the Toronto‐2005 Δ*cas3* mutant as revealed by PCR. Shown is a representative Toronto‐2005 Δ*cas3* mutant transconjugant clone, for which both LME‐1 integrated form (based on the chrF/lmeR and lmeF/chrR products) and excised form (based on the lmeF/lmeR product) were detected.

While our plasmid transformation assays suggested robust protection against LME‐1 transfer in *L. pneumophila* str. Toronto‐2005, we next asked whether we could observe strain‐to‐strain transfer of LME‐1 in the absence of this defense. Using an assay established for another *L. pneumophila* conjugative element (Flynn and Swanson, 2014), we first marked LME‐1 with a selectable marker (gentamicin resistance) in the donor strain, Murcia‐4983. We mixed these donor cells with excess (streptomycin resistant) recipient cells and after a brief co‐incubation selected for transconjugants (gentamicin‐ and streptomycin‐resistant clones) (Fig. 6A, refer to Experimental procedures section for details). Each putative transconjugant was confirmed by PCR to prevent misinterpretation of any spontaneous streptomycin‐resistant donor cells. Strikingly, while we consistently isolated LME‐1 transconjugants in an *L. pneumophila* str. Toronto‐2005 Δ*cas3* mutant (Fig. 6B), we were unable to isolate transconjugants in wild‐type (*cas3^+^*) recipients.

### LME‐1 integration into a defenseless strain occurs through a conserved *att* site

Isolating transconjugants of LME‐1 in the defenseless *L. pneumophila* str. Toronto‐2005 Δ*cas3* background provided us with an opportunity to further explore the mechanisms of LME‐1 vulnerability. Our observations of LME‐1 in RC1 (Luneberg *et al.*, 2001) and Murcia‐4983 suggested that the element utilizes a conserved 22 nt attachment site to integrate into each genome. We also identified this sequence in *L. pneumophila* str. Toronto‐2005, yet located in a completely disparate genomic neighbourhood (flanked by distinct genes) (Fig. 6C). We next asked whether this 22 nt sequence could be used by LME‐1 to integrate within this strain. Using primers designed to distinguish between the Murca‐4983 and Toronto‐2005 sequences flanking this conserved sequence (Fig. 6D), we observed integration of LME‐1 into the recipient strain's predicted attachment site (Fig. 6E). Remarkably, this palindromic sequence is conserved in every *L. pneumophila* isolate sequenced to date (Fig. S5A). Consistent with palindromic sequences being hot spots for the transposition of insertion sequence elements in other bacteria (Tobes and Pareja, 2006), we propose that, in the absence of DNA defenses, LME‐1 is capable of parasitizing a wide range of *L. pneumophila* strains through this common *att* sequence of unknown function.

### Sequence‐based LME‐1 escape from Legionella CRISPR‐Cas

**Figure 7:**
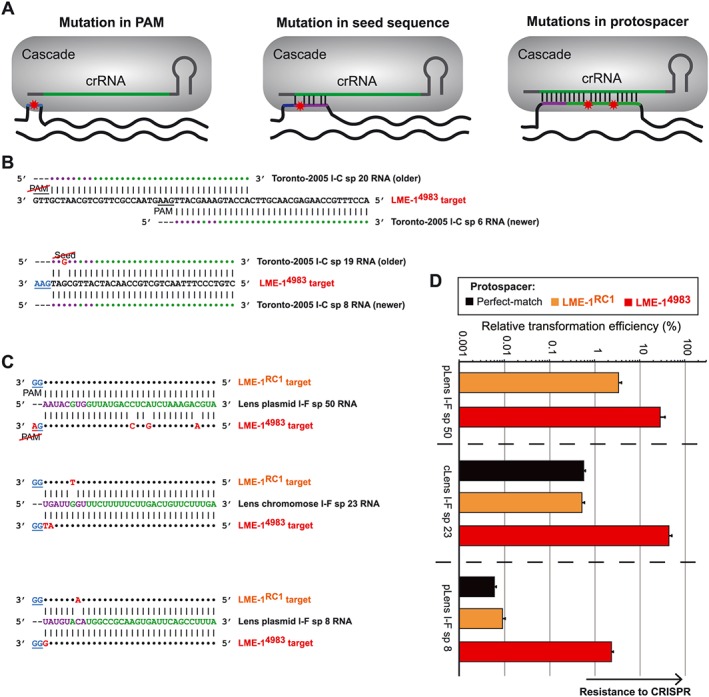
Sequence‐based LME‐1 escape from *Legionella* CRISPR‐Cas. A. Three mechanisms by which targeted sequences can escape CRISPR defense. Green indicates the spacer region in the crRNA; blue indicates the PAM sequence; purple indicates the seed sequence, and red asterisk indicates unrecognition or mismatch. B. The ST222 type I‐C CRISPR‐Cas system acquires multiple spacers that target overlapping regions in LME‐1 with differences in predicted efficiency. Shown are the LME‐1^4983^ sequences each targeted by two CRISPR spacers, of which the older spacer carries mismatches to either the PAM (underlined) or the seed sequence while the newer is a perfect match. C. LME‐1^4983^ has acquired polymorphisms that are predicted to diminish its targeting by the *Legionella pneumophila* str. Lens chromosome (c) and plasmid (p) type I‐F CRISPR‐Cas systems. For each spacer, both the LME‐1^RC1^ and LME‐1^4983^ targets are shown (with underlined protospacer adjacent sequences). Nucleotides that match the spacer crRNA are represented by dots, and those that mismatch are highlighted in red nucleotides. D. As predicted by these mismatches, LME‐1^4983^ displays markedly greater resistance than LME‐1^RC1^ to *L. pneumophila* str. Lens type I‐F CRISPR‐Cas systems. Plasmids containing protospacer targets shown in C were electroporated into *L. pneumophila* str. Lens, along with non‐targeted, scrambled controls. Black bars indicate perfectly matched protospacers; orange indicate LME‐1^RC1^ protospacers, and red indicate the LME‐1^4983^ protospacers. Note that the LME‐1^RC1^ protospacer is itself a perfect match for pLens I‐F spacer 50. The relative transformation efficiency was calculated by normalizing to the transformation efficiency of the untargeted control plasmid. Error bars indicate the standard error of the mean of three biological replicates.

Given the frequency with which *L. pneumophila* CRISPR‐Cas spacers target LME‐1, we next asked whether these genome defense systems and the invasive element might be engaged in an evolutionary arm race. One common mechanism by which other parasitic elements evade CRISPR‐Cas immunity is through frequent mutations (Deveau *et al.*, 2008; Semenova *et al.*, 2011; Cady *et al.*, 2012) (Fig. 7A). Because spacer acquisition is a directional event (Barrangou *et al.*, 2007), we asked whether newly acquired spacers in any of the strains were a better match to LME‐1 than older ones. Two pairs of CRISPR spacers in *L. pneumophila* str. Toronto‐2005 target overlapping regions of LME‐1, allowing direct comparison of predicted efficiencies between spacers acquired at different times during the evolutionary history of this strain (Fig. 7B). For each pair, the more recently acquired spacer is a perfect match to the corresponding LME‐1^4983^ sequence, whereas the older spacer is predicted to provide less protection through mutation of either the PAM or the seed sequence. One interpretation of these data is that the ancestor of *L. pneumophila* str. Toronto‐2005 was challenged by LME‐1 ‘escape’ mutants, which led to naturally primed acquisition of the newer spacers we observe.

Given their sequence divergence, we next asked if LME‐1^RC1^ and LME‐1^4983^ are equally well targeted by each *L. pneumophila* CRISPR‐Cas system. We compared the protospacer sequences in each of the two versions of LME‐1 and identified a list of polymorphisms with the potential to influence the efficiency of CRISPR targeting (Table S3). While both LME‐1^RC1^ and LME‐1^4983^ were isolated from European strains, our sequence analysis indicates that each element should be equally targeted by the diverse Ontario CRISPR‐Cas systems. Remarkably, however, we observed polymorphisms in all three LME‐1^4983^ protospacers for the two independent CRISPR‐Cas systems of one specific French strain, *L. pneumophila* str. Lens (Fig. 7C). These analyses suggested that *L. pneumophila* str. Lens might be diminished in its capacity to protect against LME‐1^4983^ because of mutations in PAM sequences, seed sequences or several other positions within each target. To test if these polymorphisms provide LME‐1^4983^ with the ability to evade *L. pneumophila* str. Lens CRISPR‐Cas, we compared the transformation efficiencies of LME‐1^RC1^ and LME‐1^4983^ protospacer‐containing plasmids. For each of the three polymorphic protospacers, *L. pneumophila* str. Lens was able to efficiently protect against LME‐1^RC1^ sequences, but the corresponding protospacers from LME‐1^4983^ were not well targeted by the CRISPR‐Cas activity of this strain (Fig. 7D). Taken together, these data point to LME‐1^4983^ having at least transiently escaped the *L. pneumophila* str. Lens CRISPR‐Cas system.

## Discussion

The historic genomic record of *L. pneumophila* CRISPR‐Cas has revealed environmental challenges faced by this important human pathogen. The record present in numerous, globally distributed *L. pneumophila* strains clearly identifies a previously obscure genetic element as a recurrent challenge faced by these pathogens. Based on our observations, we propose that LME‐1 is a potent condition‐specific modulator of the pathogen's fitness, leading to growth disadvantages in macrophages, axenic growth and at least one species of amoebal hosts, *D. discoideum*. While LME‐1 conversely leads to growth advantages in at least two other amoebae (*A. castellanii and A. polyphaga*), we and others have previously proposed that the negative fitness costs of host specialization are a key driver of *Legionella* evolution (O'Connor *et al.*, 2011; Ensminger *et al.*, 2012; Ensminger, 2016). As *L. pneumophila* cycles through over at least 15 species of diverse protozoa in the environment (Rowbotham, 1980; Fields, 1996; Solomon *et al.*, 2000; Molmeret *et al.*, 2005), acquisition of LME‐1 would likely confer a costly host range switch under environmental conditions of sufficient host diversity. Note that this could occur within freshwater, the typical habitat of *L. pneumophila* (Fliermans *et al.*, 1979), as *Dictyostelium* species have been observed in lakes and sediment (O'Dell, 1979; Richards *et al.*, 2005; Somboonna *et al.*, 2012; Shanan *et al.*, 2015); at the same time, *L. pneumophila* has also been isolated from soil (Wallis and Robinson, 2005; Schalk *et al.*, 2014; van Heijnsbergen *et al.*, 2014), a long‐established habitat of *D. discoideum* (Singh, 1947). While *D. discoideum* is generally thought of within the context of a laboratory model for *Legionella* infection, these data suggest that further scrutiny should be placed on the natural association of *L. pneumophila* and *D. discoideum* in the environment. More generally, the observation that CRISPR‐Cas effectively counters a host specialization event further supports our earlier models that host cycling (continual passage through a series of different protozoan hosts in the environment) plays a critical role in shaping numerous aspects of *L. pneumophila* evolution (O'Connor *et al.*, 2011; Ensminger *et al.*, 2012; O'Connor *et al.*, 2012).

The observation that numerous, evolutionarily diverse *L. pneumophila* strains actively defend against LME‐1 suggests that in the absence of CRISPR‐Cas, these strains would be intrinsically vulnerable to its effects. Indeed, we show that deleting *cas3* from *L. pneumophila* str. Toronto‐2005 allows for LME‐1 acquisition by this defenseless strain and integration into a conserved 22 nt sequence. Remarkably, conservation of this *att* site extends to species beyond *L. pneumophila*: *Legionella oakridgensis*, *Legionella hackeliae*, *Legionella fallonii*, *Legionella micdadei* and *Legionella longbeachae* also contain this sequence with at least 21/22 nt identity (Fig. S5B). While the presence of the 22 nt *att* site in these evolutionary distinct strains and species might represent a ‘scar’ from previous encounters with LME‐1, local sequence conservation extends to a broader 29 nt sequence across all *L. pneumophila* strains (and complete or near identity across several other species) (Fig. S5) – an observation that is less consistent with the scar hypothesis. As such, we propose a model in which the LME‐1 *att* site is a conserved genomic element of unknown, essential function, such as small non‐coding RNA (Faucher and Shuman, 2011). The intrinsic function of the LME‐1 *att* sequence in *L. pneumophila* and other species is an extremely interesting avenue of future study.

Also looking forward, *L. pneumophila* str. Toronto‐2005 holds significant promise as an experimental model to study CRISPR‐Cas adaptation. Efficient spacer acquisition has been observed in other bacteria using either phage‐mediated selection (Barrangou *et al.*, 2007), overexpression of Cas1 and Cas2 (Yosef *et al.*, 2012) or by taking advantage of the ability of inefficient targeting to prime the secondary acquisition events (Datsenko *et al.*, 2012). We observe robust spacer acquisition events through priming, which to the best of our knowledge is the first direct experimental evidence for spacer acquisition in a type I‐C CRISPR‐Cas system. Notably, priming is likely biologically relevant to *L. pneumophila* spacer acquisition. Multiple *L. pneumophila* CRISPR‐Cas arrays have multiple spacers targeting LME‐1 (Fig. 4B) and two pairs of CRISPR spacers actually target overlapping regions – one interpretation of which is that the older imperfectly targeted protospacers may have induced the acquisition of new spacers (Fig. 7). Careful observation of sequence bias and strand preference of acquired spacers in this system holds the potential to provide critical mechanistic insight into the process of adaptation, just as it has for several other CRISPR‐Cas systems (Datsenko *et al.*, 2012; Li *et al.*, 2014; Richter *et al.*, 2014; Redding *et al.*, 2015). From the limited number of laboratory‐acquired spacers that we have examined so far, we observe a mild strand preference for spacer acquisition (from the same strand as the priming sequence) but no enrichment for spacers in proximity to the priming site (Fig. 3B).

Of the 440 spacers we identified across nine evolutionarily distinct CRISPR‐Cas systems, 15 (3.4%) spacers target LME‐1 or closely related sequences. An additional 16 (3.6%) spacers target environmental sequences from metagenomic surveys. Identifying the source of those environmental protospacers and the targets of the remaining over 90% spacers is a clear priority going forward. Several lines of evidence suggest that *L. pneumophila* is challenged by the same exogenous sequence elements frequently during its normal lifestyle. First, we observe homology between multiple spacers from evolutionarily distinct CRISPR‐Cas systems (Table S4), suggesting that these distinct strains were challenged by yet another common foreign sequence. Second, within individual CRISPR arrays, we observe spacers with the same or overlapping sequence, indicative of multiple invasion of one strain by closely related foreign sequences. These observations suggest that, like LME‐1, there are likely to be other frequent mobile elements, phage or other common challenges experienced by an evolutionarily broad, geographically distributed set of strains. Ultimately, identifying and harnessing these challenges may represent a promising new strategy to prevent outbreaks and limit the severity of accidental microbial disease.

## Experimental procedures

### Bacterial strains and transgenics

*Legionella pneumophila* strain Toronto‐2005 is a clinical isolate from the 2005 outbreak of Legionnaires' disease in Toronto, Canada (Gilmour *et al.*, 2007). The other ST222 strains, Toronto‐2000, Ottawa‐2005 and Mississauga‐2006 and London‐2007, were also isolated from patients in Ontario (Tijet *et al.*, 2010). These Ontario clinical strains were kind gifts from Public Health Ontario. Philadelphia‐1 is a previously described clinical isolate from the 1976 outbreak in Philadelphia, PA, USA. The Murcia strain ST37 4288 is a clinical isolate from the 2001 outbreak in Murcia, Spain (Garcia‐Fulgueiras *et al.*, 2003). The other Murcia strains, including ST1833 4983, ST1358 4981, ST1 4683 and ST367 4774, were environmental isolates collected from different water facilities in Murcia during the outbreak period. A Δ*cas3* deletion mutant of *L. pneumophila* str. Toronto‐2005 RpsL^K43R^ was generated by allelic exchange as described (Ensminger *et al.*, 2012). To construct the pJB4648‐derived suicide plasmid, approximately 2 kb each of the flanking sequences of *cas3* was amplified and joined by PCR. The yielded colonies after sucrose selection were screened by PCR, and the *cas3* deletion was confirmed by Sanger sequencing. A LME‐1^GentR^ marked strain was generated from Murcia‐4983 using homologous recombination. Briefly, a construct was made to contain a 2 kb region from LME‐1^4983^ with an insertion of a gentamicin resistance cassette in the middle. This construct was electroporated into Murcia‐4983, and a double cross‐over recombination was selected by gentamicin. The resulting marked clone was used as donor for transferring LME‐1. Primers used in this study are available upon request.

### Genome sequencing, assembly and analysis

Custom Illumina libraries for Ontario and Murcia strains were generated as previously described (Rao *et al.*, 2013) or using Nextera XT tagmentation. Genomes were sequenced on the HiSeq 2500 and MiSeq platforms at the Donnelly Sequencing Centre at the University of Toronto. These data, including specific metrics for each library, are available from the sequence read archive, BioProject PRJNA288830. Raw paired‐end reads were *de novo* assembled using ABySS v1.3.5 (Simpson *et al.*, 2009) to generate draft genomes. The plasmid in Mississauga‐2006 was identified by the overlapping sequence at the start and the end of one *de novo* contig and further verified by PCR using primers 5′‐CAGCGCATTTTAAAGCATCA‐3′ and 5′‐GTTCGTGATAATCGCAGCAA‐3′. To circularize the *L. pneumophila* str. Toronto‐2005 genome, long‐read Pacific Biosciences sequencing was performed at Genome Quebec and assembled using Hierarchical Genome Assembly Process (Chin *et al.*, 2013). The circularized genome was further polished by reference aligning paired‐end Illumina reads using Bowtie 2 (Langmead and Salzberg, 2012) and high‐confidence SNP calling using Samtools (Li *et al.*, 2009). Methylated nucleotides across the genome (methylome) were also directly identified through Pacific Biosciences sequencing as previously described (Powers *et al.*, 2013). To minimize annotation‐based contributions to differences in perceived gene content between strains, annotations for all the finished or draft genomes were generated using Prokka (Seemann, 2014), and these annotations were used for subsequent comparisons. BLAST Ring Image Generator v0.95 (Alikhan *et al.*, 2011) was used to visualize the overall similarity between the Ontario ST222 genomes and other publicly available, finished genomes. To identify the core genome and unique genes of the indicated genomes, the Pan‐Genome Analysis Pipeline (PGAP) was used with the MultiParanoid method under default settings (e‐value < 1e‐10, coverage > 0.5, local alignment > 0.25 and global alignment > 0.5) (Zhao *et al.*, 2012). The aligned core genomes were then used to generate whole‐genome neighbour‐joining phylogenetic tree using MEGA v6.0 (Tamura *et al.*, 2013) with 500 bootstrap iterations.

### CRISPR identification and target search

CRISPR arrays in newly sequenced genomes were identified using CRISPRFinder (Grissa *et al.*, 2007). Potential targets of all the *L. pneumophila* CRISPR spacers were identified using CRISPRTarget (Biswas *et al.*, 2013), with extra weighing on the known PAM motifs: 5′‐GAA‐3′ for I‐C (Mojica *et al.*, 2009), 5′‐GG‐3′ for I‐F (Mojica *et al.*, 2009) at the 3′ region of protospacer and 5′‐CCN‐3′ for II‐B (Fonfara *et al.*, 2014) at the 5′ region of protospacer. To search the identified LME‐1 element in our strain collection, PGAP (Zhao *et al.*, 2012) was used to find homology to LME‐1 genes in selected draft genomes.

### Transformation efficiency assay

To evaluate the protective capacity of *L. pneumophila* CRISPR‐Cas systems, transformation efficiency was measured of plasmids containing either CRISPR protospacer sequence (with appropriate PAM sequence added) or scrambled control sequence (Bondy‐Denomy *et al.*, 2013). These plasmids were constructed by cloning the insert (refer to Table S5) into the ApaI/PstI‐cut pMMB207 vector (Solomon *et al.*, 2000). Electroporation was performed similarly as previously described (Rao *et al.*, 2013). Briefly, an overnight culture of *L. pneumophila* was grown from a 2‐day‐old patch in N‐(2‐Acetamido)‐2‐aminoethanesulfonic acid (ACES)‐buffered yeast extract (AYE) liquid medium at 37°C. Unless otherwise stated, bacteria were collected at post‐exponential phase (A600 nm ~4.0) to allow for a higher level of CRISPR‐Cas gene expression. Pellets of 1 ml of bacteria were washed twice with 1 ml of ice‐cold water and once with 1 ml of ice‐cold 10% glycerol and resuspended in 200 µl of ice‐cold 10% glycerol. Every 50 µl of resuspension was mixed with 200 ng of plamid DNA and transferred to an ice‐cold, 2 mm gap electroporation cuvette (VWR). Electroporation was performed on an ECM 630 (BTX) electroporator set to 600 Ω, 25 mF, 2.5 kV. After 4~5 h recovery in AYE medium, the electroporated cells were plated in dilution series onto charcoal‐buffered ACES yeast extract (CYE) plates supplemented with 5 µg ml^−1^ of chloramphenicol and incubated at 37°C for 4 days. The relative transformation efficiency for each target plasmid was calculated as a percentage of the transformation efficiency obtained for the control plasmid. *L. pneumophila* str. Philadelphia‐1 was used in each set of experiment to control for the amount of transformed plamid DNA.

### Axenic passage in broth for defined generations

An overnight culture of bacteria was grown to exponential phase (OD_600_ of 2.0) and back diluted to OD_600_ of 0.0625. Cultures were grown at 37°C, shaking in a 48‐well plate (Greiner). Using a Tecan M200 Pro plate reader connected to a Freedom EVO 100 liquid handler, every 20 min, the optical density was measured of each well. Once OD_600_ reached 2.0, cultures were back diluted 32‐fold to the next wells containing fresh AYE medium and continued growth. In this way, cultures passaged for every five generations were transferred and saved in a separate 48‐well plate kept at 4°C.

### Detection of spacer acquisition

The pMMB207 plasmids containing either protospacer sequence or scrambled sequence were electroporated into *L. pneumophila* str. Toronto‐2005 RpsL^K43R^ to evaluate primed or ‘naive’ spacer acquisition respectively. The transformed clones were passaged in broth without antibiotic selection to allow plasmid loss. An aliquot of resulting cultures were used as template to PCR amplify the leader end of the CRISPR array. The resulting products were separated on 2% agarose gels. Higher molecular weight bands indicate spacer acquisition and were purified, Topo‐cloned and Sanger sequenced. Additional spacers were obtained by aligning the sequences to the original CRISPR array.

### Bacterial infection of various host cells

TPA‐differentiated THP‐1 macrophages were seeded at 1 × 10^7^ cells in 10 ml of RPMI 1640 supplemented with glutamine and 10% heat‐inactivated fetal bovine serum in a T75 flask (CELLSTAR) and incubated at 37°C with 5% CO_2_. *Acanthamoeba castellanii* (ATCC‐30234) and *A. polyphaga* (ATCC‐30461) were plated at 1 × 10^7^ cells in 3 ml of Ac buffer (Ensminger and Isberg, 2010) in a 6‐well plate (Greiner) and maintained at 37°C. *Vermamoeba (Hartmannella) vermiformis* (ATCC‐50237) was plated at 1 × 10^7^ cells in 3 ml of *V. vermiformis* medium (modified proteose‐peptone, yeast extract, yeast nucleic acid, folic acid, and hemin (PYNFH), ATCC medium 1034 supplemented with 1 mg/L hemin) in 6‐well plate (Greiner) and maintained at 35°C. *Dictyostelium discoideum* (strain ID DBS0236176 – derived from AX2) was plated at 1.5 × 10^7^ cells in 10 ml of MB medium {20 mM MES [2(N‐morpholino)ethanesulfonic acid) (pH 6.9), 0.7% yeast extract, 1.4% BBL thiotone E peptone} (Solomon *et al.*, 2000) in 92 mm petri dish and maintained at 25.5°C. An overnight culture of bacteria was grown to post‐exponential phase (motile and OD_600_ between 4.0 and 5.0) and inoculated to the host cells at an multiplicity of infection of 0.01. Infections were harvested at 72 h post inoculation. To lyse the host cells, THP‐1 cells were incubated in 10 ml of water; amoebae were passaged five times through 27G needles. Host debris was removed from the lysates by low‐speed centrifugation (400×g for 5 min). Bacteria were subsequently collected from the supernatant by high‐speed centrifugation (7000×g for 15 min). The resulting bacteria pellets were washed once with 1 ml of water and genomic DNA extracted using the NucleoSpin Tissue kit (Machery‐Nagel). The extracted genomic DNA was then used in qPCR to measure LME‐1 episomal frequency.

#### Quantitative PCR analysis

The qPCR reactions were carried out using the SensiFAST SYBR Hi‐ROX Kit (Bioline) on the StepOnePlus Real‐Time PCR platform (Applied Biosystems). To measure the transcriptional level of CRISPR‐Cas genes, mRNA was extracted from bacteria grown to exponential phase (A600 nm ~2.0) or post‐exponential phase (A600 nm ~4.0) using the PureLink RNA Mini Kit (Life Technologies). The mRNA was then reverse transcribed into cDNA using the SuperScript VILO cDNA Synthesis Kit (Life Technologies). In this set of qPCR experiment, genomic DNA was used in the standard curve and the 16S rRNA gene was used as an internal reference. To measure the frequency of the episomal LME‐1, a set of primer pairs were used to amplify from the genomic DNA extracted from bacteria before or after infection. In this set of qPCR experiment, plasmids containing each amplification product were used in the standard curve. The LME‐1 excision frequency and episomal copy number are calculated as

$$\begin{array}{l} \text{Freq}\left(\text{Excision}\right)=\frac{\text{Amplicon}\;\left(\text{chrF}\&\text{chrR}\right)}{\text{Amplicon}\;\left(\text{chrF}\&\text{chrR}\right)+\left[\text{Amplicon}\left(\text{chrF}\&\text{lmeR}\right)+\text{Amplicon}\left(\text{lmeF}\&\text{chrR}\right)\right]/2}\\ \text{CopyNumber}\left(\text{Episome}\right)=\frac{\text{Amplicon}\left(\text{lmeR}\&\text{lmeF}\right)}{\text{Amplicon}\left(\text{chrF}\&\text{chrR}\right)} \end{array}$$

#### Transfer of LME‐1

To analyse the protection of CRISPR‐Cas against LME‐1 transfer, the LME‐1‐marked Murcia‐4983 strain (Gent^R^, Strep^S^) was used as donor, and either *L. pneumophila* str. Toronto‐2005 RpsL^K43R^ (*cas3* ^+^) or its derivative Δ*cas3* strain (Gent^S^, Strep^R^) was used as recipient. Transfer experiments were performed following the conjugation protocol as described (Flynn and Swanson, 2014). Specifically, both the donor and the recipient were cultured to post‐exponential phase and were mixed on 0.22 µm filters placed on pre‐warmed CYE agar plates, with over 10‐fold excessive amount of recipient cells in the mixture. After 2 h incubation at 37°C, the mating mixtures were resuspended in water and plated on CYE plates supplemented with 15 µg ml^−1^ of gentamicin and 50 µg ml^−1^ of streptomycin to select for transconjugants. To distinguish between true transconjugant and spontaneous Strep^R^ mutant of donor, the resulting Gent^R^ Strep^R^ colonies were screened by the presence or absence of PCR product specific to donor, recipient or LME‐1. Integration of LME‐1 to the chromosomal *att* site in the true transconjugant was confirmed by PCR using primers for LME‐1 and primers for Toronto‐2005‐specific sequences flanking the *att* site.

#### Data accessibility

GenBank accession numbers for the Toronto‐2005 complete genome and LME‐1^4983^ sequence are CP012019 and KT271770 respectively. Raw Illumina reads for each previously uncharacterized strain described in the paper, including *L. pneumophila* str. Toronto‐2005, Toronto‐2000, Ottawa‐2005, Mississauga‐2006, London‐2007, Murcia‐2001‐4288, Murcia‐2001‐4683, Murcia‐2001‐4774, Murcia‐2001‐4983 and Murcia‐2001‐4981, are deposited in the NCBI Sequence Read Archive under the BioProject PRJNA288830.

## Author contributions

CR and AWE conceived of the experiments and comparative genomic analyses that CR performed subsequently. CG and CP provided strains and participated in discussions. JBD assisted with CRISPRTarget searches and provided guidance on PAM selection for the transformation efficiency assays. JW and KD supervised Pacific Biosciences sequencing and subsequent downstream analysis of the Toronto‐2005 genome and methylome. CR and AWE wrote the manuscript, and the other authors contributed to its revision.

## Supporting information


**Fig. S1.** Schematics of the full‐length *rtxA* gene and the two mobile elements in *L. pneumophila* str. Toronto‐2005. A. Shown is the structure of the full‐sequence *rtxA* gene in *L. pneumophila* str. Toronto‐2005. The repetitive units are identified using Tandem repeats finder (Benson, 1999). B and C. Shown are the predicted genes in the *tra* and *lvh* region encoding for each type of proteins as indicated by different colours. Genes unique to *L. pneumophila* str. Toronto‐2005 are highlighted by a black border. Note that two type I R–M systems are identified as ‘cargo’ genes in the *lvh* region. Potential *att* sequences are identified flanking the two regions, each with one site overlapping or adjacent to a non‐coding RNA gene.
**Fig. S2.** Ontario ST222 strains have a highly similar genome and share a type I‐C CRISPR‐Cas system. A. Shown is the genome ring map generated using BLAST Ring Image Generator v0.95 (Alikhan *et al.*, 2011). The five ST222 strains from Ontario are highly similar except for the two mobile elements (*tra* and *lvh*). Note that the type I‐C CRISPR‐Cas system is conserved in all these ST222 strains, while the type I R–M *a* system in the *lvh* region only exists in the two 2005 strains. B. Schematics of the plasmid‐borne type I‐F CRISPR‐Cas system in *L. pneumophila* str. Mississauga‐2006. C. Core genome‐based neighbour‐joining phylogenetic tree of the five ST222 strains. Note that the genomes phylogeny is consistent with the derivative relationship of the ST222 strains predicted from the type I‐C CRISPR arrays (Fig. 2A).
**Fig. S3.** Characterization of the type I‐C CRISPR‐Cas activity. A. Shown are the relative transcriptional levels of the type I‐C CRISPR‐Cas system under different bacterial growth phase. The mRNA levels of indicated pre‐crRNA fragment or *cas* genes were measured by qPCR using the cDNA prepared from overnight culture of the Toronto‐2005 strain harvested at either exponential (grey bars) or post‐exponential (black bars) phase. The 16S rRNA was used as internal control. Error bars indicate the standard error of the mean of three biological replicates, and shown is one representative of two independent experiments. B. Protection efficiencies of a list of spacers on the Toronto‐2000 CRISPR array. Plasmids containing protospacers matching the indicated spacers (for indexes, refer to Fig. 2A) were electroporated into the indicated strains. The relative transformation efficiency was calculated by normalizing to the transformation efficiency of the control plasmid that contains an untargeted sequence. Error bars indicate the standard error of the mean of three biological replicates.
**Fig. S4.** Gradual loss of a CRISPR‐targeted plasmid during axenic passage. *Legionella pneumophila* str. Toronto‐2005 and the derivative Δ*cas3* strain transformed with the targeted plasmid pSp1 were passaged in AYE broth in the absence of antibiotic selection for defined generations. Cultures of each time point were plated onto selective (CYE + chloramphenicol) and non‐selective (CYE) plates to measure maintenance of pSp1 during passage. Error bars indicate the standard error of the mean of three independent clones, and this plot is representative of two separate experiments.
**Fig. S5.** *Legionella* commonly harbors a short, conserved sequence that may confer susceptibility to LME‐1 integration. A. A conserved intragenic palindromic sequence is present in numerous *Legionella pneumophila* strains and can be exploited as an attachment (*att*) site by LME‐1 to integrate into the bacterial chromosome. Shown is the schematic of this *att* site and the adjacent regions in various *L. pneumophila* strains. Orthologous genes are indicated by arrows of the same colour. B. The 22 nt *att* site is present in several other species of *Legionella*. Shown is the schematic of the genomic context of the *att* sequence in strains from different species, with each annotated gene (not orthologous) represented by a grey arrow. Note that the *att* sequence in *Legionella micdadei* is identified in a putative prophage region (Gomez‐Valero *et al.*, 2014). In both A and B, the zoomed‐in alignment of the *att* sequence region in different strains is shown, with the 22 nt *att* sequence in *L. pneumophila* str. Murcia‐4983 highlighted in red box and the broader 29 nt conserved sequence marked in grey background.
**Table S1.** List of *L. pneumophila* CRISPR spacers. A. List of CRISPR spacers identified from sequenced *L. pneumophila* strains. B. List of acquired spacers during axenic passage under priming conditions.
**Table S2**. Summary of target hits of all available *L. pneumophila* CRISPR spacers.
**Table S3**. Summary of CRISPR target hits in two versions of LME‐1.
**Table S4**. Summary of homologous *L. pneumophila* CRISPR spacers.
**Table S5**. Primer information, including inserts used in transformation assays.
**Table S6**. qPCR measurements of episomal frequency.

Supporting info item
