# Supplementary material for: Active and adaptive Legionella CRISPR‐Cas reveals a recurrent challenge to the pathogen
Source: Cell Microbiol. 2016 Mar 31;18(10):1319–38. doi: 10.1111/cmi.12586 (PMC5071653; doi:10.1111/cmi.12586)
Supplement: Supplementary file 1 — Supporting info item [file CMI-18-1319-s001.zip › Supporting Information - Legends.pdf]

## SUPPORTING INFORMATION:

**Fig. S1. Schematics of the full-length *rtxA* gene and the two mobile elements in *L. pneumophila* str. Toronto-2005.**

**A.** Shown is the structure of the full-sequence *rtxA* gene in *L. pneumophila* str. Toronto-2005. The repetitive units are identified using Tandem repeats finder (Benson, 1999). **B and C.** Shown are the predicted genes in the *tra* and *lvh* region encoding for each type of proteins as indicated by different colors. Genes unique to *L. pneumophila* str. Toronto-2005 are highlighted by a black border. Note that two type I R-M systems are identified as "cargo" genes in the *lvh* region. Potential *att* sequences are identified flanking the two regions, each with one site overlapping or adjacent to a non-coding RNA gene.

**Fig. S2. Ontario ST222 strains have a highly similar genome and share a type I-C CRISPR-Cas system.**

**A.** Shown is the genome ring map generated using BLAST Ring Image Generator (BRIG) v0.95 (Alikhan *et al.*, 2011). The five ST222 strains from Ontario are highly similar except for the two mobile elements (*tra* and *lvh*). Note that the type I-C CRISPR-Cas system is conserved in all these ST222 strains, while the type I R-M *a* system in the *lvh* region only exists in the two 2005 strains. **B.** Schematics of the plasmid-borne type I-F CRISPR-Cas system in *L. pneumophila* str. Mississauga-2006. **C.** Core-genome based Neighbor-joining phylogenetic tree of the five ST222 strains. Note that the genomes phylogeny is consistent with the derivative relationship of the ST222 strains predicted from the type I-C CRISPR arrays (Fig. 2A).

**Fig. S3. Characterization of the type I-C CRISPR-Cas activity.**

**A.** Shown are the relative transcriptional levels of the type I-C CRISPR-Cas system under different bacterial growth phase. The mRNA levels of indicated pre-crRNA fragment or *cas* genes were measured by qPCR using the cDNA prepared from overnight culture of the Toronto-2005 strain harvested at either exponential (grey bars) or post-exponential (black bars) phase. The 16S rRNA was used as internal control. Error bars indicate the standard error of the mean of three biological replicates, and shown is one representative of two independent experiments. **B.** Protection efficiencies of a list of spacers on the Toronto-2000 CRISPR array. Plasmids containing protospacers matching the indicated spacers (for indexes, see Fig. 2A) were electroporated into the indicated strains. The relative transformation efficiency was calculated by normalizing to the transformation efficiency of the control plasmid that contains an untargeted sequence. Error bars indicate the standard error of the mean of three biological replicates.

**Fig. S4. Gradual loss of a CRISPR-targeted plasmid during axenic passage**

*L. pneumophila* str. Toronto-2005 and the derivative Δ*cas3* strain transformed with the targeted plasmid pSp1 were passaged in AYE broth in the absence of antibiotic selection for defined generations. Cultures of each time point were plated onto selective (CYE + chloramphenicol) and non-selective (CYE) plates to measure maintenance of pSp1 during passage. Error bars indicate the standard error of the mean of three independent clones, and this plot is representative of two separate experiments.

**Fig. S5. *Legionella* commonly harbors a short, conserved sequence that may confer susceptibility to LME-1 integration.**

**A.** A conserved intragenic palindromic sequence is present in numerous *L. pneumophila* strains and can be exploited as an attachment (*att*) site by LME-1 to integrate into the bacterial chromosome. Shown is the schematic of this *att* site and the adjacent regions in various *L. pneumophila* strains. Orthologous genes are indicated by arrows of the same color. **B.** The 22 nt *att* site is present in several other species of *Legionella*. Shown is the schematic of the genomic context of the *att* sequence in strains from different species, with each annotated gene (not orthologous) represented by a grey arrow. Note that the *att* sequence in *L. micdadei* is identified in a putative prophage region (Gomez-Valero *et al.*, 2014). In both **A** and **B,** the zoomed-in alignment of the *att* sequence region in different strains is shown, with the 22 nt *att* sequence in *L. pneumophila* str. Murcia-4983 highlighted in red box, and the broader 29 nt conserved sequence marked in grey background.

**Table S1. List of *L. pneumophila* CRISPR spacers.**

**A.** List of CRISPR spacers identified from sequenced *L. pneumophila* strains. **B.** List of acquired spacers during axenic passage under priming conditions.

**Table S2. Summary of target hits of all available *L. pneumophila* CRISPR spacers.**

**Table S3. Summary of CRISPR target hits in two versions of LME-1.**

**Table S4. Summary of homologous *L. pneumophila* CRISPR spacers.**

**Table S5. Primer information, including inserts used in transformation assays.**

**Table S6. qPCR measurements of episomal frequency.**
