# Supplementary material for: Active and adaptive Legionella CRISPR‐Cas reveals a recurrent challenge to the pathogen
Source: Cell Microbiol. 2016 Mar 31;18(10):1319–38. doi: 10.1111/cmi.12586 (PMC5071653; doi:10.1111/cmi.12586)
Supplement: Supplementary file 1 — Supporting info item [file CMI-18-1319-s001.zip › Table-S2.pdf]

**Table S2. Summary of target hits of all available *L. pneumophila* CRISPR spacers.**

| *L. pneumophila* spacer | CRISPRTarget hit* | Score** | Note |
|---|---|---|---|
| Toronto-2005-I-C_spacer6 | Legionella pneumophila serogroup 1, 30 kb instable genetic element(AJ277755) position: 19398-19364 | 39 | |
| Toronto-2005-I-C_spacer8 | Legionella pneumophila serogroup 1, 30 kb instable genetic element(AJ277755) position: 19082-19115 | 39 | |
| Toronto-2005-I-C_spacer7 | Legionella pneumophila serogroup 1, 30 kb instable genetic element(AJ277755) position: 16793-16828 | 39 | |
| Murcia-2001-ST1358_spacer4 | Legionella pneumophila serogroup 1, 30 kb instable genetic element(AJ277755) position: 13779-13746 | 39 | |
| Toronto-2005-I-C_spacer19 | Legionella pneumophila serogroup 1, 30 kb instable genetic element(AJ277755) position: 19082-19115 | 37 | |
| Mississauga-2006-I-F_spacer59 | Legionella pneumophila serogroup 1, 30 kb instable genetic element(AJ277755) position: 27925-27956 | 37 | |
| Mississauga-2006-I-F_spacer12 | Legionella pneumophila serogroup 1, 30 kb instable genetic element(AJ277755) position: 19949-19918 | 37 | |
| Lens-Plasmid-I-F_spacer50 | Legionella pneumophila serogroup 1, 30 kb instable genetic element(AJ277755) position: 22663-22632 | 37 | |
| Lens-Plasmid-I-F_spacer32 | Activated sludge metagenome contig16020, whole genome shotgun sequence(AERA01015926) position: 92-61 | 37 | |
| 130b-II-B_spacer18 | Legionella pneumophila str. Paris complete genome(CR628336) position: 191030-190999 | 37 | target in Paris CRISPR array |
| Paris-II-B_spacer1 | Activated sludge metagenome contig28417, whole genome shotgun sequence(AERA01027227) position: 676-639 | 37 | |
| Mississauga-2006-I-F_spacer74 | Legionella pneumophila serogroup 1, 30 kb instable genetic element(AJ277755) position: 20793-20762 | 35 | |
| Lens-Chromosome-I-F_spacer23 | Legionella pneumophila serogroup 1, 30 kb instable genetic element(AJ277755) position: 2286-2255 | 35 | |
| Lens-Plasmid-I-F_spacer8 | Legionella pneumophila serogroup 1, 30 kb instable genetic element(AJ277755) position: 22464-22433 | 35 | |
| Toronto-2005-I-C_spacer40 | Bioreactor metagenome contig_8387, whole genome shotgun sequence(AMWB02161224) position: 8364-8400 | 32 | |
| Lens-Plasmid-I-F_spacer42 | Legionella pneumophila 230099 Alcoy, complete genome(CP001828) position: 1660145-1660176 | 31 | |
| Lens-Plasmid-I-F_spacer42 | Legionella pneumophila str. Corby, complete genome(CP000675) position: 1661616-1661647 | 31 | |
| Alcoy-I-F_spacer32 | Uncultured Gokushovirinae clone WSBWG10n1 major capsid protein gene, partial cds(KF689311) position: 642-673 | 31 | |
| Toronto-2005-I-C_spacer40 | marine metagenome genome assembly TARA_109_DCM_0.22, contig TARA_109_DCM_<-0.22_C7530914_1, whole geno | 30 | |
| Toronto-2005-I-C_spacer40 | Stromatolite metagenome 36158673, whole genome shotgun sequence(ABMT01027850) position: 35-69 | 30 | |
| Toronto-2005-I-C_spacer40 | Marine metagenome 1096626615272, whole genome shotgun sequence(AACY020460958) position: 2985-2951 | 30 | |
| Toronto-2005-I-C_spacer40 | Leptospira borgpetersenii serovar Pomona str. 200901868 ctg1130573510788, whole genome shotgun sequence(NZ_AKWF( | 30 | |
| 130b-II-B_spacer17 | Hypersaline lake metagenome ctg7180000052828, whole genome shotgun sequence(APHM01003927) position: 998-965 | 30 | |
| Mississauga-2006-I-F_spacer6 | Marine metagenome 27717628, whole genome shotgun sequence(ABMN01350822) position: 37-67 | 29 | |
| Alcoy-I-F_spacer28 | marine metagenome genome assembly TARA_122_DCM_0.1-0.22, contig TARA_122_DCM_0.1-0.22_C4943966_1, whole | 29 | |
| Lens-Plasmid-I-F_spacer4 | Marine metagenome 1096626097875, whole genome shotgun sequence(AACY023989113) position: 624-653 | 29 | |
| Alcoy-I-F_spacer20 | Atta colombica fungus garden Top 2030384248, whole genome shotgun sequence(AGFS01078387) position: 526-495 | 29 | |
| Lens-Plasmid-I-F_spacer7 | Activated sludge metagenome contig06523, whole genome shotgun sequence(AERA01006474) position: 469-500 | 29 | |
| Lens-Plasmid-I-F_spacer6 | Uncultured marine Microviridae clone SOG3-01 major capsid protein gene, partial cds(KC131005) position: 118-144 | 29 | |
| Murcia-2001-ST1358_spacer29 | AC013759 Homo sapiens chromosome 18, clone RP11-2E13, complete sequence(AC013759) position: 78162-78195 | 29 | |
| Paris-II-B_spacer30 | Schistocephalus solidus genome assembly S_solidus_NST_G2 ,scaffold SSLN_contig0000006(LL901847) position: 26226-2 | 29 | |
| Murcia-2001-ST1358_spacer31 | Arcobacter butzleri 7h1h, complete genome(CP006615) position: 474871-474902 | 29 | |
| Toronto-2005-I-C_spacer40 | PREDICTED: Apis mellifera uncharacterized LOC102654118 (LOC102654118), ncRNA(XR_409015) position: 97-73 | 28 | |
| Toronto-2005-I-C_spacer9 | Legionella pneumophila serogroup 1, 30 kb instable genetic element(AJ277755) position: 23822-23789 | 28 | |
| Mississauga-2006-I-F_spacer7 | Marine metagenome ctg_1101667006744, whole genome shotgun sequence(AACY022599393) position: 453-426 | 27 | |
| Lens-Chromosome-I-F_spacer35 | Paenibacillus sp. FSL H7-0357, complete genome(CP009241) position: 1285752-1285725 | 27 | |
| Paris-II-B_spacer11 | PREDICTED: Dasypus novemcinctus coiled-coil-helix-coiled-coil-helix domain containing 7 (CHCHD7), transcript variant | 27 | |
| Murcia-2001-ST1358_spacer19 | PREDICTED: Amphimedon queenslandica prolyl endopeptidase, transcript variant 2 (PREP), mRNA(XM_003387403) posi | 26 | |
| Toronto-2005-I-C_spacer40 | PREDICTED: Jaculus jaculus armadillo repeat containing, X-linked 1 (Armcx1), mRNA(XM_004668776) position: 1677-16 | 24 | |
| Murcia-2001-ST1358_spacer11 | Marine metagenome 1096626651460, whole genome shotgun sequence(AACY020488987) position: 3133-3158 | 22 | |

* Only non-redundant targets with score>20 and the right PAM are listed here.

** PAM match score +5
