# Supplementary material for: Active and adaptive Legionella CRISPR‐Cas reveals a recurrent challenge to the pathogen
Source: Cell Microbiol. 2016 Mar 31;18(10):1319–38. doi: 10.1111/cmi.12586 (PMC5071653; doi:10.1111/cmi.12586)
Supplement: Supplementary file 1 — Supporting info item [file CMI-18-1319-s001.zip › Table-S3.pdf]

Table S3. Summary of CRISPR target hits in two versions of LME-1.

***L. pneumophila* spacer**	**Target in RC1 LME-1**	**Target in Murcia-4983 LME-1**	**Any polymorphism between RC1 and 4983 targets**	**Note**
Toronto-2005-I-C_spacer6	1 mismatch at position 36	perfect match	Position 36 silent SNP	
Toronto-2005-I-C_spacer7	1 mistmatch at position 19	1 mistmatch at position 19	/	
Toronto-2005-I-C_spacer9	6 mismatches at position 3,6,7,11,30,35	5 mismatches at position 3,5,6,7,30	Position 5,6 nonsynonymous SNPs, Position 11,35 silent SNPs	
Toronto-2005-I-C_spacer8	perfect match	perfect match	/	
Toronto-2005-I-C_spacer19	1 mistmatch at position 3	1 mistmatch at position 3	/	overlap with Toronto-2005-I-C_spacer8
Toronto-2005-I-C_spacer20	perfect match, but wrong PAM	perfect match, but wrong PAM	/	overlap with Toronto-2005-I-C_spacer6
Mississauga-2006-I-F_spacer12	perfect match	perfect match	/	
Mississauga-2006-I-F_spacer59	perfect match	perfect match	/	
Mississauga-2006-I-F_spacer74	1 mismatch at position 25	perfect match	Position 25 nonsynonymous SNP	
Lens-Chromosome-I-F_spacer23	1 mismatch at position 6	2 mismatches at position 1,2	Position 1,2 nonsynonymous SNP, Position 6 silent SNP	
Lens-Plasmid-I-F_spacer8	1 mismatch at position 7	1 mistmatch at position 1	Position 1,7 silent SNPs	
Lens-Plasmid-I-F_spacer50	perfect match	PAM mutated, 3 mismatches at position 17,20,29	Position 17,20,29 silent SNPs	
Alcoy-I-F_spacer34	wrong PAM, 4 mismatches at position 9,21,26,30	wrong PAM, 4 mismatches at position 9,21,26,30	/	
Alcoy-I-F_spacer36	No target	perfect match		in 4983 LME-1 unique region
Murcia-2001-ST1358-II-B_spacer4	Perfect match	Perfect match	/	
