# Supplementary material for: Active and adaptive Legionella CRISPR‐Cas reveals a recurrent challenge to the pathogen
Source: Cell Microbiol. 2016 Mar 31;18(10):1319–38. doi: 10.1111/cmi.12586 (PMC5071653; doi:10.1111/cmi.12586)
Supplement: Supplementary file 1 — Supporting info item [file CMI-18-1319-s001.zip › Table-S4.pdf]

Table S4. Summary of homologous *L. pneumophila* CRISPR spacers.

Query	Subject	evalue	alignment length	Identity %	mismatches	gaps	q.start	q.end	s.start	s.end	bit score
Toronto-2005-I-C_spacer8	Toronto-2005-I-C_spacer19	2.00E-16	31	100	0	0	4	34	4	34	61.9
Toronto-2005-I-C_spacer12	Toronto-2005-I-C_spacer28	9.00E-19	35	100	0	0	1	35	1	35	69.9
Toronto-2005-I-C_spacer22	Lens-Plasmid-I-F_spacer12	3.00E-12	32	93.75	2	0	1	32	32	1	48.1
Mississauga-2006-I-F_spacer4	Mississauga-2006-I-F_spacer8	5.00E-17	32	100	0	0	1	32	1	32	63.9
Mississauga-2006-I-F_spacer47	Lens-Plasmid-I-F_spacer49	1.00E-14	32	96.88	1	0	1	32	1	32	56
Mississauga-2006-I-F_spacer57	Mississauga-2006-I-F_spacer72	1.00E-11	31	93.55	2	0	1	31	2	32	46.1
Mississauga-2006-I-F_spacer60	Lens-Chromosome-I-F_spacer58	2.00E-16	31	100	0	0	1	31	2	32	61.9
Mississauga-2006-I-F_spacer66	Mississauga-2006-I-F_spacer67	5.00E-17	32	100	0	0	1	32	1	32	63.9
Lens-Plasmid-I-F_spacer22	Lens-Plasmid-I-F_spacer47	2.00E-07	32	87.5	4	0	1	32	1	32	32.2
Lens-Plasmid-I-F_spacer26	Murcia-2001-ST367-II-B_spacer11	7.00E-10	28	92.86	2	0	5	32	32	5	40.1
Lens-Plasmid-I-F_spacer41	Alcoy-I-F_spacer3	1.00E-11	27	96.3	1	0	1	27	1	27	46.1
Lens-Plasmid-I-F_spacer44	Paris-II-B_spacer19	2.00E-16	31	100	0	0	2	32	34	4	61.9
Paris-II-B_spacer1	130b-II-B_spacer1	1.00E-20	38	100	0	0	1	38	1	38	75.8
Paris-II-B_spacer1	Murcia-2001-ST367-II-B_spacer1	1.00E-20	38	100	0	0	1	38	1	38	75.8
Paris-II-B_spacer20	130b-II-B_spacer18	6.00E-17	32	100	0	0	1	32	1	32	63.9
Murcia-2001-ST1358-II-B_spacer14	Murcia-2001-ST1358-II-B_spacer17	3.00E-18	34	100	0	0	1	34	1	34	67.9
