# Supplementary material for: Active and adaptive Legionella CRISPR‐Cas reveals a recurrent challenge to the pathogen
Source: Cell Microbiol. 2016 Mar 31;18(10):1319–38. doi: 10.1111/cmi.12586 (PMC5071653; doi:10.1111/cmi.12586)
Supplement: Supplementary file 1 — Supporting info item [file CMI-18-1319-s001.zip › Table-S6.pdf]

Table S6

Episomal frequency (%) in AYE	
Mean	1.00
SEM	0.17
Median	0.79
Min	0.41
Max	2.19
Biological replicates	12

Episomal copy
Mean
SEM
Median
Biological replicates

	Host		
	D. discoideum	**THP-1**	***V. vermiformis***
**Input frequency (%)***	1.93	0.74	0.74
**Input SEM**	0.17	0.07	0.07
**Output frequency (%)**	0.41	0.39	0.98
**Output SEM**	0.05	0.00	0.08
**Fold-change (mean)**	0.21	0.53	1.35
**Fold-change (SEM)**	0.02	0.05	0.21

*** THP-1, *V. vermiformis*, and *A. castellanii* infections were done on the same day.**
(3 biological replicates per experiment)

/ number
221.33
15.50
226.29
3

A. castellanii	***A. polyphaga***
0.74	0.51
0.07	0.05
2.47	5.27
0.28	0.92
3.34	10.21
0.25	1.08
