# Supplementary figures and images for: Active and adaptive Legionella CRISPR‐Cas reveals a recurrent challenge to the pathogen
Source: Cell Microbiol. 2016 Mar 31;18(10):1319–38. doi: 10.1111/cmi.12586 (PMC5071653; doi:10.1111/cmi.12586)

Figure S1

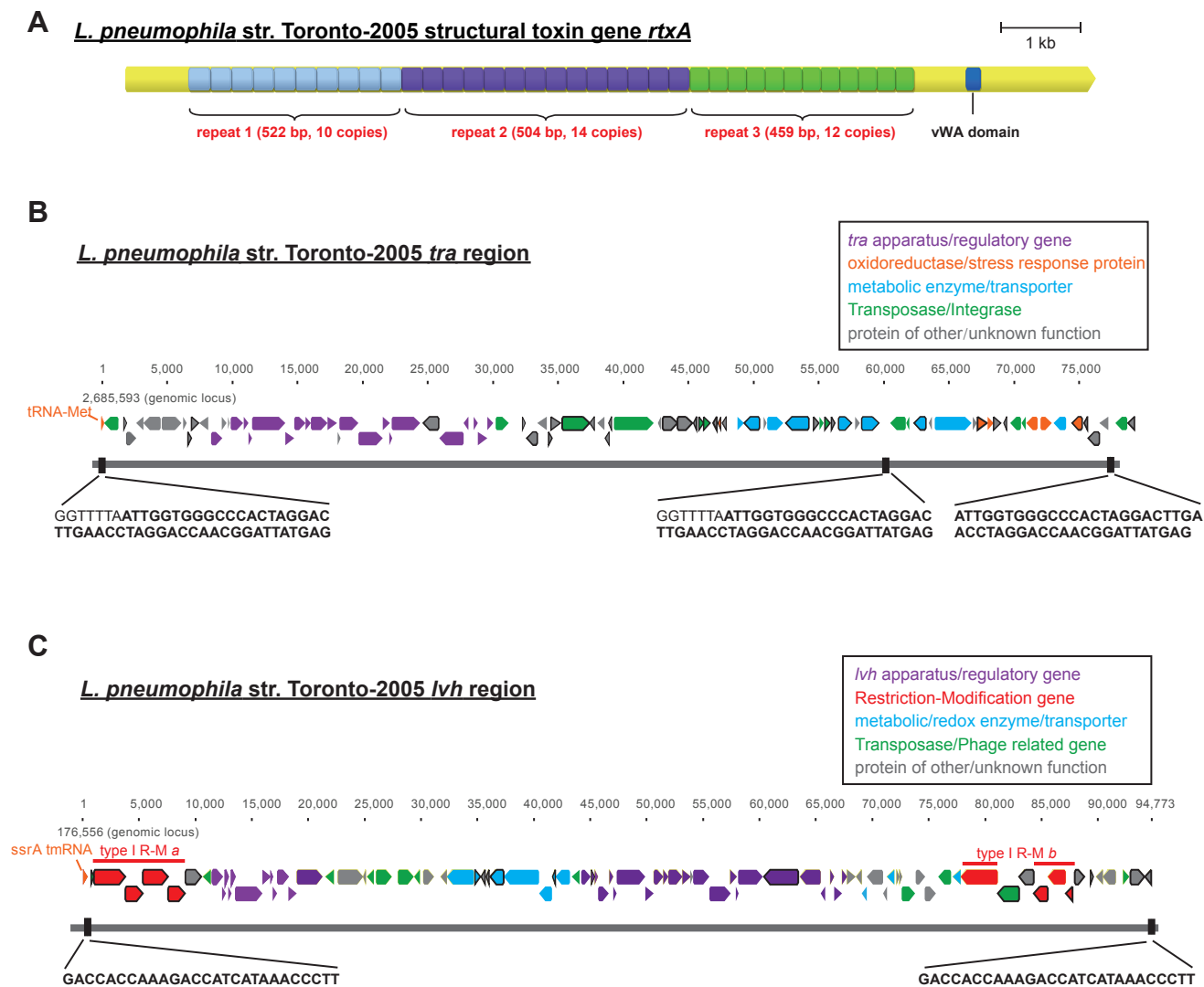

Supplement: Supplementary file 1 — Supporting info item [file CMI-18-1319-s001.zip › Figure-S1.pdf]

Figure S2

A

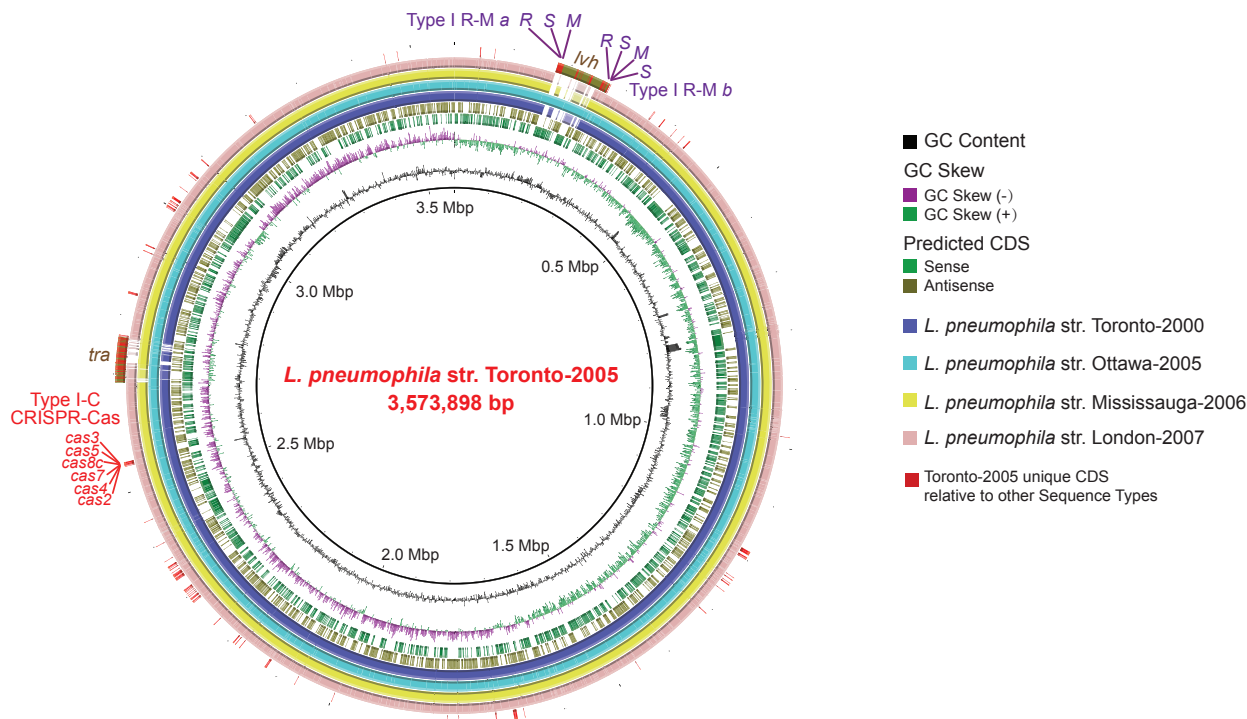

B

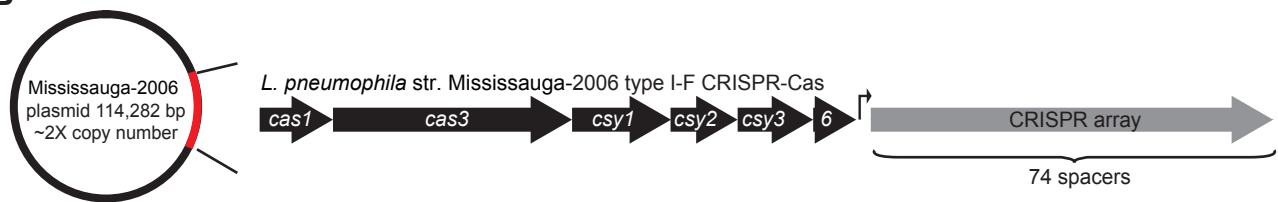

C

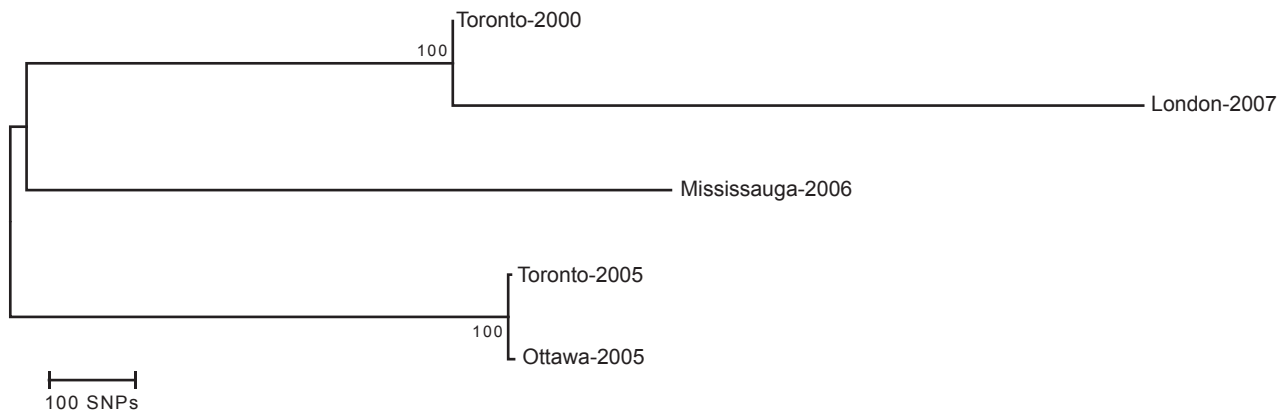

Supplement: Supplementary file 1 — Supporting info item [file CMI-18-1319-s001.zip › Figure-S2.pdf]

Figure S3

A

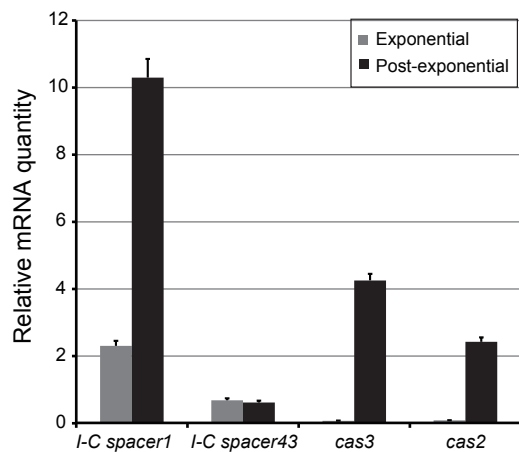

B

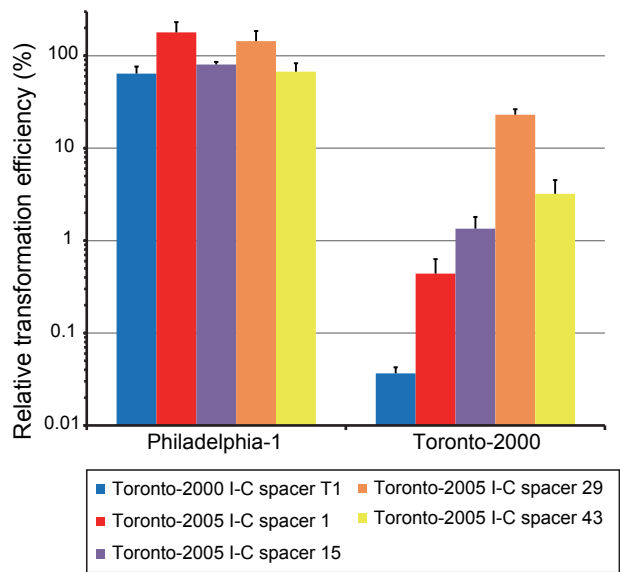

Supplement: Supplementary file 1 — Supporting info item [file CMI-18-1319-s001.zip › Figure-S3.pdf]

**Figure S4**

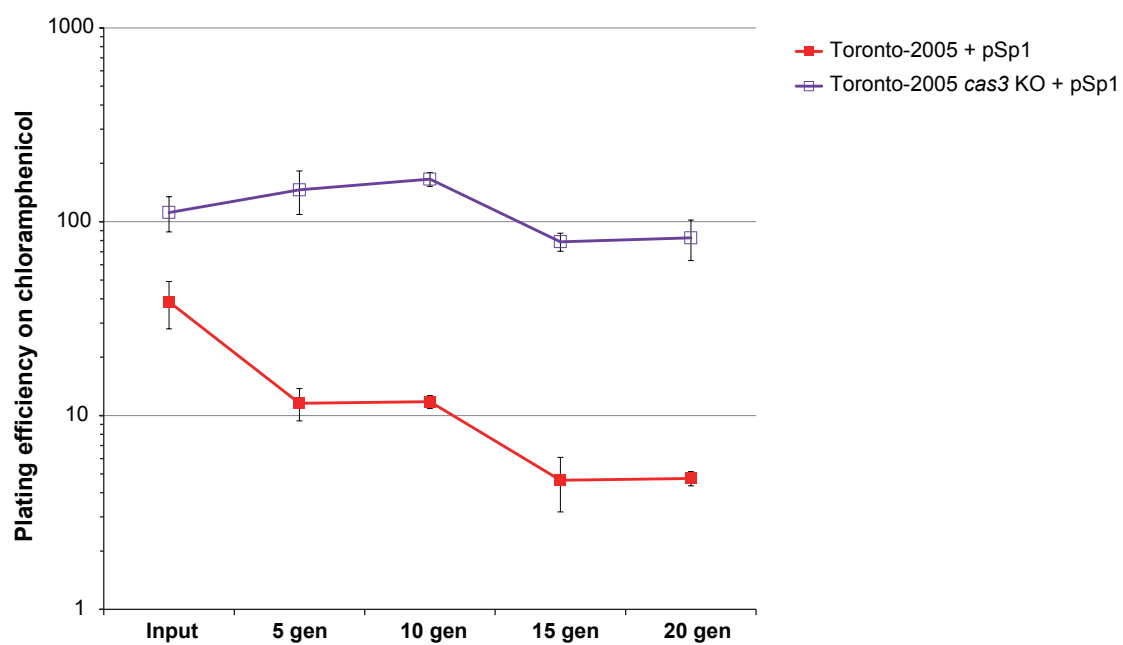

Supplement: Supplementary file 1 — Supporting info item [file CMI-18-1319-s001.zip › Figure-S4.pdf]

Figure S5

A

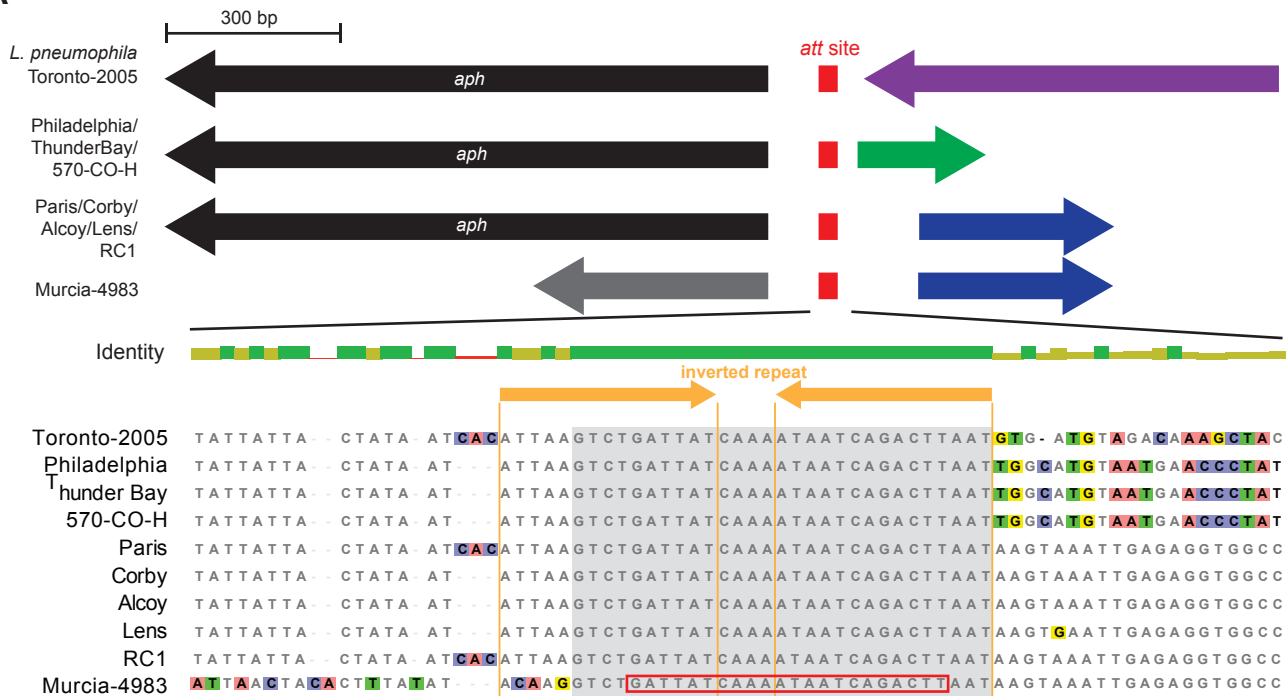

B

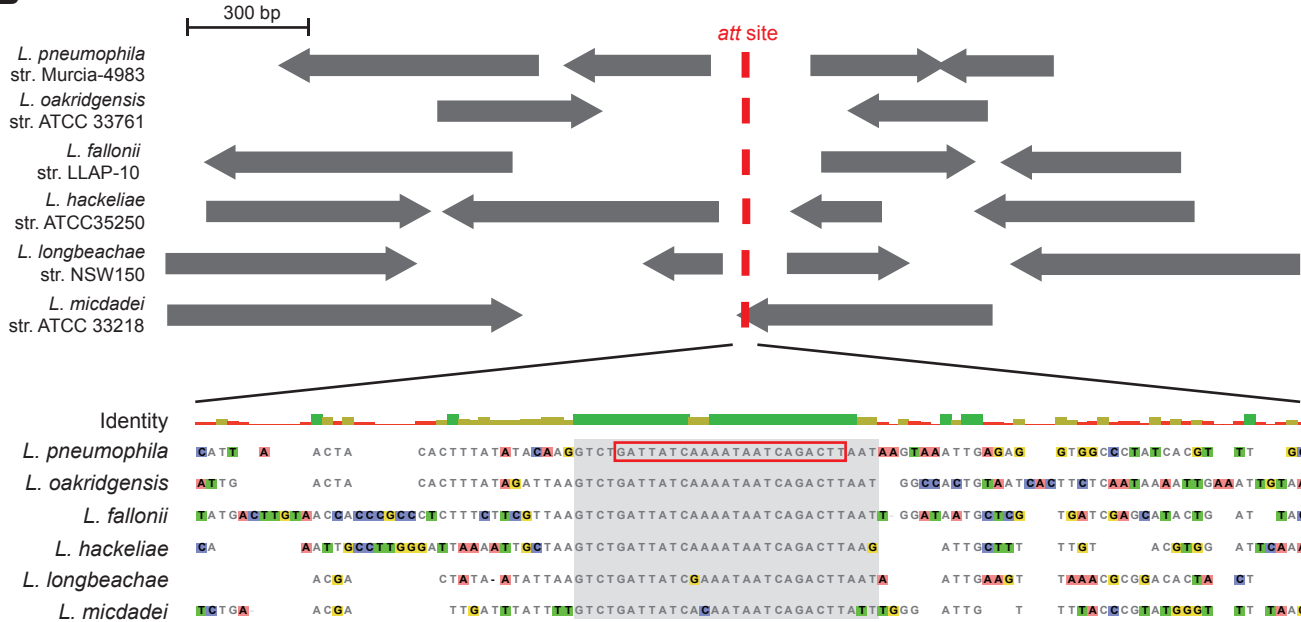

Supplement: Supplementary file 1 — Supporting info item [file CMI-18-1319-s001.zip › Figure-S5.pdf]
